# Association of Genetic and Environmental Risks for Attention-Deficit/Hyperactivity Disorder With Hypomanic Symptoms in Youths

**DOI:** 10.1001/jamapsychiatry.2019.1949

**Published:** 2019-08-14

**Authors:** Georgina M. Hosang, Paul Lichtenstein, Angelica Ronald, Sebastian Lundström, Mark J. Taylor

**Affiliations:** 1 Centre for Psychiatry, Wolfson Institute of Preventive Medicine, Barts and the London School of Dentistry and Medicine, Queen Mary, University of London, London, United Kingdom; 2 Department of Medical Epidemiology & Biostatistics, Karolinska Institutet, Stockholm, Sweden; 3 Department of Psychological Science, Birkbeck, University of London, London, United Kingdom; 4 Gillberg Neuropsychiatry Centre, University of Gothenburg, Gothenburg, Sweden; 5 Sweden Centre for Ethics, Law and Mental Health, University of Gothenburg, Gothenburg, Sweden

## Abstract

**Importance:**

Attention-deficit/hyperactivity disorder (ADHD) and bipolar disorder are highly comorbid, with significantly associated symptoms. The mechanisms that account for their co-occurrence are not known.

**Objective:**

To examine the degree to which genetic and environmental risk factors for ADHD traits, across childhood and adolescence, are associated with adolescent hypomanic symptoms.

**Design, Setting, and Participants:**

This study used data on 13 532 twin pairs from the Child and Adolescent Twin Study in Sweden, a prospective, longitudinal twin study. Their parents provided ADHD data when children were 9 or 12 years of age. Of those who reached 15 years of age, 3784 participated. Of those who reached 18 years of age, 3013 participated. The study was performed from December 20, 2017, to December 5, 2018. Data analysis was performed at the Department of Medical Epidemiology & Biostatistics, Karolinska Institutet, Stockholm, Sweden, from March 1, 2018, to October 31, 2018.

**Main Outcomes and Measures:**

Attention-deficit/hyperactivity disorder traits and hypomanic symptoms were assessed using parent-rated instruments. Associations between ADHD and adolescent hypomanic symptoms across childhood and adolescence were investigated using generalized estimating equations. Multivariate twin models were used to examine the extent to which genetic and environmental risk factors for ADHD were associated with hypomania.

**Results:**

Among 3784 15-year-old twin pairs and 3013 18-year-old twin pairs, ADHD and hypomanic symptoms were significantly associated (age 15 years: β = 0.30; 95% CI, 0.24-0.34; *P* < .001; age 18 years: β = 0.19; 95% CI, 0.16-0.22; *P* < .001), especially for the hyperactivity-impulsivity ADHD symptom domain (age 15 years: β = 0.53; 95% CI, 0.46-0.60; *P* < .001; age 18 years: β = 0.36; 95% CI, 0.30-0.42; *P* < .001) compared with the inattention domain (age 15 years: β = 0.40; 95% CI, 0.34-0.47; *P* < .001; age 18 years: β = 0.24; 95% CI, 0.19-0.29; *P* < .001). Between 13% and 29% of the genetic risk factors for hypomania were also associated with ADHD, with higher estimates detected for symptoms of hyperactivity-impulsivity (10%-25%) compared with inattention (6%-16%). Environmental factors played a negligible role in the associations. Genetic factors unique to adolescent hypomania were associated with 25% to 42% of its variance, suggesting some etiologic distinction between these forms of psychopathology.

**Conclusions and Relevance:**

More than a quarter of the genetic risk factors for adolescent hypomanic traits were also associated with ADHD symptoms in childhood and adolescence, with hypomania-specific genetic risk factors detected. These findings suggest that ADHD and hypomanic symptoms are associated with shared genetic factors, which should be the focus of further research.

## Key Points

**Question:**

Do symptoms of attention-deficit/hyperactivity disorder and hypomania share genetic and environmental risk factors in young people?

**Findings:**

In this twin study of 13 532 Swedish twin pairs aged 9 and 12 years at enrollment, up to 29% of the variance for hypomanic symptoms was associated with genetic risk factors shared with attention-deficit/hyperactivity disorder traits, with differing estimates detected for hyperactivity-impulsivity (up to 25%) compared with inattention (up to 16%) attention-deficit/hyperactivity disorder symptom domains.

**Meaning:**

Attention-deficit/hyperactivity disorder and hypomanic symptoms appear to be associated with similar genetic factors.

## Teaser

This twin study examines the degree to which genetic and environmental risk factors for attention-deficit/hyperactivity disorder (ADHD) traits, across childhood and adolescence, are associated with adolescent hypomanic symptoms.

## Introduction

Early identification of individuals at high risk of bipolar disorder (BD) is essential for prevention and intervention^1^ and can be aided by investigating hypomanic symptoms in youths. Bipolar disorder is characterized by hypomanic or manic episodes. Hypomania is common in youths, with up to 10% identified as being at high risk of BD based on the clustering, duration, and impairment of symptoms.^2^ Subsyndromal hypomanic symptoms or traits have been linked to subsequent manic or hypomanic episodes and BD onset.^3^

There is evidence that BD is preceded by childhood ADHD; a population-based study^4^ found higher incidence rates of BD among those with a history of ADHD (incidence rate, 23.86) compared with those without (incidence rate, 2.17). The comorbidity rates of BD-ADHD are higher than expected by chance^5^; the weighted mean prevalence of ADHD in pediatric bipolar samples is 48%.^6^ A study^7^ that used modified BD diagnostic criteria characterized by nonepisodic irritability and ultrarapid cycling in pediatric samples reported high ADHD-BD comorbidity rates of 74% to 98%. The focus on nonepisodic irritability, which also covers temper outbursts and emotion dysregulation, may have increased these comorbidity rates because such symptoms are associated features of ADHD.^7,8^ After much debate, there is some consensus that nonepisodic irritability is more characteristic of severe mood dysregulation or disruptive dysregulation disorder rather than BD.^8^ Thus, care needs to be taken to differentiate between BD and these syndromes.

Significant, modest correlations between adolescent hypomanic and hyperactivity symptoms have also been reported.^2,9^ The ADHD symptom domains of hyperactivity-impulsivity and inattention may be differentially associated with BD. One study^10^ found that BD was associated with inattentive and combined but not hyperactive-impulsive ADHD presentations. Others^11,12^ have reported similar levels of inattention and hyperactivity-impulsivity in patients with BD. These disparate findings need to be addressed with further research. The co-occurrence of ADHD and BD is associated with worse outcomes, including higher rates of comorbidity and suicide attempts, compared with BD or ADHD alone.^11,12,13^ It is crucial to determine the origins of the ADHD-BD overlap, distinguishing inattention from hyperactivity-impulsivity, to avoid such outcomes.

Shared genetic risk factors are postulated to be partly responsible for the ADHD-BD association and their related symptoms. A moderate genetic correlation between ADHD and BD II using family data has been reported.^14^ That study^14^ focused on BD not hypomania and did not distinguish between the ADHD presentations. The sample had a broad age range; thus, it is unclear whether the results apply to different age groups. Research focused on childhood and adolescence would be useful because the initial emergence of psychopathologic symptoms occurs at these developmental stages. This was the first twin study, to our knowledge, to explore the extent to which genetic and environmental risk factors for hypomanic traits are associated with ADHD symptoms in youths, examining inattention and hyperactivity-impulsivity separately.

## Methods

### Participants

This twin study used data from 13 532 twin pairs who participated in the Child and Adolescent Twin Study in Sweden, a longitudinal, prospective twin study performed from December 20, 2017, to December 5, 2018.^15^ Their parents provided ADHD data when children were 9 or 12 years of age (response rate, 75%; 3951 monozygotic [MZ] twins and 9581 dizygotic [DZ] twins). Of those who reached 15 years of age, 3784 participated (response rate of those eligible, 61%; 1115 MZ twins and 2669 DZ twins). Of those who reached 18 years or older, 3013 participated (response rate of those eligible, 59%; 983 MZ twins and 2030 DZ twins). Pairs were excluded if either twin had a known brain injury or chromosomal disorder (n = 207). Data analysis was performed at the Department of Medical Epidemiology & Biostatistics, Karolinska Institutet, Stockholm, Sweden, from March 1, 2018, to October 31, 2018. Parents provided consent for themselves and their children to participate at 9 or 12 years of age, and the twins and their parents gave separate written informed consent at subsequent waves after receiving the study description. The study was approved by the Karolinska Institutet Ethical Review Board. All data were deidentified.

### Measures

Hypomania was assessed at 15 years of age using the parent-rated Child Mania Rating Scale (CMRS),^16^ which distinguishes children with BD from children with ADHD and healthy control individuals with high sensitivity (sensitivity for children with ADHD, 0.84; sensitivity for controls, 0.90) and specificity (specificity for children with ADHD, 0.92; specificity for controls, 0.96).^16^ The parent-rated Mood Disorders Questionnaire^17^ was used to assess hypomanic symptoms at 18 years of age, with high sensitivity (sensitivity, 0.72) and specificity (specificity, 0.81) in identifying adolescent BD.^17^ Both instruments cover symptoms that are more specific to mania (eg, hypersexuality and grandiosity) compared with other forms of psychopathology (ADHD).^18^ Further details of all measures are presented in eTable 1 in the Supplement.

The ADHD symptoms at 9 and 12 years of age were assessed using the Autism-Tics, ADHD, and Other Comorbidities Inventory (A-TAC),^19^ a structured telephone interview completed by parents. The A-TAC ADHD domain consists of impulsivity and activity as well as concentration and attention modules, corresponding to the *DSM-IV* ADHD criteria.^19^ Parents rated ADHD symptoms when the twins were 15 years of age using the Strengths and Difficulties Questionnaire hyperactivity subscale,^20^ and the ADHD *DSM-IV* subscale of the Adult Behavior Checklist^21^ was used when twins were 18 years of age. The Adult Behavior Checklist was divided into hyperactivity-impulsivity (6 items) and inattention (7 items) domains based on similarity with *DSM-5* criteria.

Participants who had received an ADHD (*International Statistical Classification of Diseases and Related Health Problems, Tenth Revision (ICD-10)* code, F90) and/or BD diagnosis (*ICD-10* codes, F30-F31) were identified using the Swedish National Patient Register,^22^ which records all specialist inpatient and outpatient care given to residents of Sweden. Cases of BD were also identified through lithium prescriptions using the Prescribed Drug Register,^23^ which covers all medications prescribed to residents of Sweden since 2005.

###  Statistical Analysis

Positively skewed variables were log-transformed. The birth year associations were included as a covariate in all twin analyses, and means were permitted to differ by sex to account for mean sex differences. Participants were split into groups based on published cutoffs for the hypomania (CMRS: score of ≥10; Mood Disorders Questionnaire: minimum of ≥7 symptoms clustered in same period with at least moderate impairment) and ADHD (A-TAC: broad cutoff: ≥6; strict cutoff: ≥12) instruments (eTable 1 in the Supplement). In separate analyses, participants who received an ADHD and/or BD diagnosis in the Swedish National Patient Register (and/or a prescription of lithium in the Prescribed Drug Register for BD) were compared with those without such diagnoses. To test whether ADHD traits at each age (number of symptoms, screening diagnoses, and clinical diagnoses) were associated with hypomanic traits at 15 and 18 years of age, linear regressions within generalized estimating equations (GEEs) were performed with ADHD as the exposure and hypomania the outcome. This approach allows for clustering of related individuals and calculates robust SEs. To assess the associations between each ADHD diagnosis and high risk of BD, we implemented logistic regressions that calculated odds ratios within a GEE framework, adjusting for sex and birth year. The GEEs were implemented in the drgee package of R.^24^ A 1-sided *P* < .05 was considered to be statistically significant.

### Twin Analyses

The classic twin method was used to investigate the degree to which genetic and environmental risk factors for ADHD traits were associated with hypomanic symptoms. This method relies on comparing the correlations between MZ twins who share all their segregating DNA code and DZ twins who share approximately 50% of their segregating DNA code. On the basis of this information, variance in and among phenotypes can be decomposed into additive genetic risk factors (A), nonadditive genetic risk factors (D), common or shared environmental risk factors (C; common to both twins and increase their similarity), and nonshared or unique environmental risk factors (E; related to environmental factors that differ across twins, including measurement error).^25^ The general principles of the twin design are described in detail elsewhere.^25^ Cross-trait cross-twin correlations involve correlating one twin’s ADHD score with their co-twin’s hypomania score; by calculating these correlations separately for MZ and DZ twins, the degree to which genetic and environmental factors affect the covariance between ADHD and hypomania can be estimated. The eMethods in the Supplement give the analytic codes used in the analyses.

**Figure 1.**
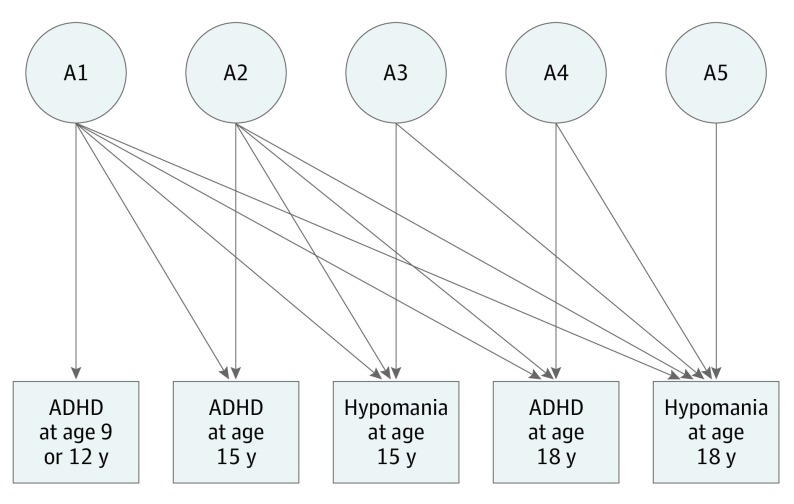
Path Diagram for Cholesky Decomposition The pathways from the latent variables (enclosed in circles) labeled A1, A2, and A4 to the 2 hypomania scales estimate the proportion of variation in hypomania that is associated with genetic risk factors for attention-deficit/hyperactivity disorder (ADHD) traits at 9, 12, 15, and 18 years of age. The pathway from A3 to hypomania at 15 years of age represents the proportion of genetic variance in hypomania at 15 years of age that is independent of ADHD traits. Hypomania at 18 years of age was associated with genetic risk factors for ADHD traits at each age (A1, A2, and A4), genetic risk factors for hypomania at 15 years of age (A3), and genetic factors that are unique to hypomania at 18 years of age (A5). Equivalent pathways are included for C, D, and E.

Univariate models were used to assess the relative contribution of A, D, C, and E to each measure and to test assumptions of the twin design. We assessed whether genetic and environmental risk factors for ADHD traits are associated with hypomania by fitting a multivariate Cholesky decomposition to the data (Figure 1). The proportion of variance in each trait that was associated with A, D, C, and E was estimated. Variance in hypomania that is associated with genetic risk factors for ADHD was also estimated (the pathways from latent variables A1, A2, and A4 to the hypomania scales in Figure 1). The pathway from variable A3 to hypomania at 15 years of age represents the proportion of genetic variance in hypomania at 15 years of age that was independent of ADHD traits. Hypomania at 18 years of age is associated with genetic risk factors for ADHD traits (A1, A2, and A4), genetic risk factors are associated with hypomania at 15 years of age (A3), and genetic risk factors unique to hypomania at 18 years of age (A5). Equivalent pathways are included for C, D, and E. Squaring these pathways gives the proportion of variance in each trait accounted for by each pathway.^26^

Because univariate analysis implicated C for hypomania and not ADHD, we fitted a model in which A, D, and E were associated with ADHD and A, C, and E were associated with hypomania (C and D did not contribute to the covariance among phenotypes in this model), as well as the A, C, and E and A, D, and E models. Whenever D was estimated, sibling interaction paths were also included in the model because these factors can mimic that of D with twin correlations and are implicated when the assumption of equal variances across zygosity is not met. Each model was fitted with separate variance and covariance components by sex (quantitative sex limitation). The significance of these sex differences was tested by constraining all pathways to be equal by sex. We tested further nested models to assess the significance of individual groups of variance and covariance components. Model fit was assessed using Bayesian information criteria, which outperforms alternative fit statistics when fitting multivariate models to large samples. Lower Bayesian information criteria values indicate better fitting models.^27^ All analyses were repeated for both ADHD dimensions. The Strengths and Difficulties Questionnaire hyperactivity scale was omitted from these analyses because it only covers hyperactivity.

## Results

A total of 13 532 twin pairs (3951 MZ twins, 9581 DZ twins, 2031 female MZ pairs, 1920 male MZ pairs, 22231 female DZ pairs, 2582 male DZ pairs, and 4778 opposite-sex DZ pairs) participated in this study at 9 or 12 years of age, with 3784 followed up at 15 years of age and 3013 at 18 years or older. The descriptive statistics by sex and zygosity are presented in Table 1. The results of the GEEs testing the association between ADHD and hypomania traits are presented in Table 2. Symptoms of ADHD were significantly associated with hypomania at 15 years of age (β = 0.30; 95% CI, 0.26-0.34) and 18 years of age (β = 0.19; 95% CI, 0.16-0.22) after adjustment for sex and birth year. Removal of hypomania items that were similar to ADHD items (2 of 10 CMRS items and 5 of 13 Mood Disorders Questionnaire items) did not affect the results (eTable 2 in the Supplement). All diagnostic definitions of ADHD were significantly associated with being at high risk of BD (Table 2). The rates of clinically diagnosed ADHD among the 52 individuals with a formal diagnosis of BD and/or a prescription of lithium (37%) were significantly higher than that among controls (4%) (odds ratio, 15.41; 95% CI, 8.55-27.76; *P* < .001).

**Table 1.** ADHD and Hypomania Measures by Sex and Zygosity

Variable	Mean (SD)									Score Range
	Total	Male	Female	MZM	DZM	MZF	DZF	DZOS		
								Male	Female	
**ADHD Assessments**										
Age, y										
9 or 12	2.02 (2.97)	2.47 (3.42)	1.56 (2.61)	2.24 (3.21)	2.55 (3.51)	1.44 (2.43)	1.79 (2.90)	2.56 (3.48)	1.46 (2.47)	0-19
15	1.83 (1.84)	2.11 (1.94)	1.57 (1.71)	1.99 (1.82)	2.08 (1.95)	1.45 (1.62)	1.75 (1.85)	2.23 (2.01)	1.51 (1.64)	0-8
18	2.15 (2.97)	2.43 (3.17)	1.88 (2.74)	2.14 (2.78)	2.41 (3.20)	1.72 (2.50)	2.12 (3.05)	2.75 (3.46)	1.84 (2.68)	0-24
**ADHD Subscales**										
Age of 9 or 12 y										
Hyperactivity-impulsivity	0.97 (1.67)	1.17 (1.85)	0.77 (1.44)	1.09 (1.74)	1.21 (1.91)	0.70 (1.32)	0.87 (1.60)	1.19 (1.86)	0.74 (1.39)	0-10
Inattention	1.05 (1.75)	1.30 (1.93)	0.79 (1.50)	1.16 (1.79)	1.34 (1.98)	0.74 (1.43)	0.92 (1.63)	1.37 (1.98)	0.72 (1.41)	0-9
Age of 18 y										
Hyperactivity-impulsivity	0.73 (1.40)	0.76 (1.43)	0.70 (1.37)	0.65 (1.24)	0.81 (1.50)	0.60 (1.22)	0.82 (1.54)	0.80 (1.52)	0.69 (1.35)	0-12
Inattention	1.42 (1.91)	1.68 (2.07)	1.19 (1.72)	1.49 (1.86)	1.60 (2.03)	1.11 (1.61)	1.31 (1.86)	1.95 (2.27)	1.15 (1.68)	0-13
**Hypomania Measures**										
Age, y										
15	1.80 (2.43)	1.60 (2.32)	2.00 (2.53)	1.47 (2.16)	1.66 (2.43)	1.93 (2.37)	2.07 (2.69)	1.66 (2.32)	1.99 (2.52)	0-24
18	0.93 (1.85)	0.91 (1.86)	0.95 (1.85)	0.79 (1.68)	0.93 (1.90)	0.90 (1.75)	0.95 (1.90)	0.99 (1.97)	1.01 (1.92)	0-13

Abbreviations: ADHD, attention-deficit/hyperactivity disorder; DZF, dizygotic female twin pairs; DZM, dizygotic male twin pairs; DZOS, dizygotic opposite-sex twin pairs; MZF, monozygotic female twin pairs; MZM, monozygotic male twin pairs.

**Table 2.** Associations Between ADHD and Hypomania

Variable	β (95% CI)^a^	SE	1-Sided *P* Value
**Association Among ADHD Traits, Diagnoses, and Hypomania**			
Hypomania age 15 y			
ADHD symptoms	0.30 (0.24-0.34)	0.02	<.001
Hyperactive-impulsive symptoms	0.53 (0.46-0.60)	0.04	<.001
Inattentive symptoms	0.40 (0.34-0.47)	0.03	<.001
Broad cutoff ADHD^b^	2.26 (1.90-2.61)	0.18	<.001
Strict cutoff ADHD^c^	3.68 (2.42-4.95)	0.65	<.001
ADHD diagnosis^d^	3.18 (2.55-3.81)	0.32	<.001
Hypomania at age 18 y			
ADHD symptoms	0.19 (0.16-0.22)	0.02	<.001
Hyperactive-impulsive symptoms	0.36 (0.30-0.42)	0.03	<.001
Inattentive symptoms	0.24 (0.19-0.29)	0.03	<.001
Broad cutoff ADHD^b^	1.57 (1.24-1.90)	0.17	<.001
Strict cutoff ADHD^c^	2.54 (1.68-3.39)	0.44	<.001
ADHD diagnosis^d^	2.88 (2.19-3.56)	0.35	<.001
**Association Between Hypomanic Symptoms and ADHD**			
ADHD at age 15 y			
Hypomanic symptoms	1.49 (1.34-1.64)	0.08	<.001
ADHD at age 18 y			
Hypomanic symptoms	1.98 (1.79-2.17)	0.10	<.001
**Association Between ADHD Diagnoses and High Risk of Bipolar Disorder**^e^			
**Variable**	**Comparison Group/High-risk Group, No. (%)**	**Adjusted OR (95% CI)**^**a**^	**1-Sided *P* Value**
Hypomania at age 15 y			
Broad cutoff ADHD^a^	429 (0.07)/36 (38)	8.27 (5.43-12.58)	<.001
Strict cutoff ADHD^b^	56 (0.009)/12 (13)	15.66 (8.02-30.59)	<.001
ADHD diagnosis^c^	165 (0.03)/28 (29)	15.75 (9.81-25.29)	<.001
Hypomania at age 18 y			
Broad cutoff ADHD^a^	273 (0.06)/27 (43)	11.95 (7.00-20.40)	<.001
Strict cutoff ADHD^b^	36 (0.008)/7 (11)	15.46 (6.50-36.73)	<.001
ADHD diagnosis^c^	78 (0.02)/20 (32)	28.53 (15.95-51.04)	<.001

Abbreviations: ADHD, attention-deficit/hyperactivity disorder; BD, bipolar disorder; OR, odds ratio.

^a^ Adjusted for sex and age.

^b^ Broad ADHD: score of 6 or more on the Autism-Tics, ADHD, and Other Comorbidities Inventory ADHD module at 9 and 12 years of age (n = 465 [7%] at 15 years of age; n = 300 [7%] at 18 years of age).

^c^ Strict ADHD: score of 12.5 or more on the Autism-Tics, ADHD, and Other Comorbidities Inventory ADHD module at 9 and 12 years of age (n = 68 [1%] at 15 years of age; n = 43 [1%] at 18 years of age).

^d^ ADHD diagnosis: at least 1 recorded diagnosis of ADHD in the National Patient Register (n = 193 [3%] at 15 years of age; n = 98 [2%] at 18 years of age).

^e^ These data are given as number (percentage) of comparison group (n = 6301)/high-risk BD group (n = 96). High risk of BD was defined as a cutoff of 10 or more on the parent-rated Child Mania Rating Scale at 15 years of age or a parent-rated Mood Disorders Questionnaire score of at least 7 with symptoms clustered together in the same period and moderate to severe problems (eg, work and legal problems).

The continuous measures of ADHD and hypomanic symptoms were used for the remaining analyses. Twin correlations for ADHD and hypomanic traits are given in Table 3; the univariate twin analyses and fit statistics are presented in eTable 3 and eTable 4 in the Supplement. The assumption of equal means and variances across zygosity was not met for ADHD, suggesting sibling contrast pathways (eTable 3 in the Supplement). Moderate to strong heritability was found for ADHD (heritability, 0.51-0.74) and hypomania traits (heritability, 0.50-0.64).

**Table 3.** Phenotypic, Twin, and Cross-Trait Cross-Twin Correlations for ADHD and Hypomania

Variable		*r*_ph_						
		Male	Female	MZM	DZM	MZF	DZF	DZOS
**Cross-Twin Correlations**								
Age at ADHD symptoms onset, y								
9		NA	NA	0.68 (0.66 to 0.70)	0.24 (0.21 to 0.28)	0.64 (0.61 to 0.66)	0.24 (0.20 to 0.27)	0.32 (0.29 to 0.34)
15		NA	NA	0.55 (0.50 to 0.59)	0.12 (0.05 to 0.18)	0.56 (0.52 to 0.60)	0.07 (0.01 to 0.13)	0.14 (0.09 to 0.19)
18		NA	NA	0.53 (0.45 to 0.50)	0.24 (0.14 to 0.33)	0.52 (0.45 to 0.59)	0.17 (0.08 to 0.26)	0.22 (0.15 to 0.29)
Age at hyperactivity-impulsivity onset, y								
9 or 12		NA	NA	0.69 (0.66 to 0.71)	0.19 (0.15 to 0.22)	0.65 (0.62 to 0.67)	0.19 (0.16 to 0.23)	0.24 (0.22 to 0.27)
18		NA	NA	0.39 (0.28 to 0.48)	0.14 (0.04 to 0.23)	0.49 (0.41 to 0.56)	0.08 (to 0.02 to 0.17)	0.12 (0.04 to 0.19)
Age at inattention onset, y								
9 or 12		NA	NA	0.60 (0.57 to 0.63)	0.14 (0.10 to 0.17)	0.54 (0.51 to 0.56)	0.12 (0.08 to 0.15)	0.20 (0.17 to 0.23)
18		NA	NA	0.52 (0.44 to 0.59)	0.19 (0.09 to 0.29)	0.48 (0.40 to 0.55)	0.05 (to 0.05 to 0.14)	0.19 (0.12 to 0.26)
15		NA	NA	0.78 (0.75 to 0.81)	0.55 (0.49 to 0.60)	0.77 (0.74 to 0.80)	0.51 (0.45 to 0.57)	0.52 (0.48 to 0.56)
18				0.61 (0.54 to 0.67)	0.29 (0.19 to 0.37)	0.66 (0.60 to 0.70)	0.42 (0.33 to 0.50)	0.30 (0.23 to 0.37)
**Cross-Trait Cross-Twin Correlations Between ADHD and Hypomania at Age 15 y**								
Age at ADHD symptom onset, y								
9 or 12	0.28 (0.24 to 0.31)		0.32 (0.29 to 0.36)	0.26 (0.22 to 0.30)	0.07 (0.02 to 0.12)	0.26 (0.22 to 0.30)	0.13 (0.08 to 0.18)	0.14 (0.10 to 0.17)
15	0.43 (0.40 to 0.43)		0.45 (0.42 to 0.47)	0.36 (0.31 to 0.39)	0.16 (0.15 to 0.21)	0.36 (0.32 to 0.39)	0.19 (0.14 to 0.24)	0.17 (0.14 to 0.21)
18	0.39 (0.34 to 0.44)		0.38 (0.33 to 0.42)	0.31 (0.24 to 0.37)	0.20 (0.13 to 0.28)	0.36 (0.30 to 0.41)	0.21 (0.13 to 0.28)	0.19 (0.14 to 0.25)
Age at hyperactivity-impulsivity onset, y								
9 or 12	0.28 (0.25 to 0.32)		0.30 (0.27 to 0.34)	0.28 (0.23 to 0.32)	0.06 (0.05 to 0.11)	0.26 (0.22 to 0.30)	0.11 (0.06 to 0.16)	0.12 (0.09 to 0.15)
18	0.38 (0.33 to 0.43)		0.39 (0.34 to 0.44)	0.29 (0.21 to 0.36)	0.19 (0.12 to 0.27)	0.33 (0.28 to 0.39)	0.15 (0.07 to 0.23)	0.17 (0.11 to 0.23)
Age at inattention onset, y								
9	0.23 (0.19 to 0.26)		0.27 (0.24 to 0.31)	0.20 (0.15 to 0.25)	0.10 (0.04 to 0.15)	0.20 (0.16 to 0.25)	0.13 (0.08 to 0.18)	0.11 (0.07 to 0.15)
18	0.33 (0.27 to 0.38)		0.30 (0.25 to 0.35)	0.27 (0.20 to 0.34)	0.19 (0.11 to 0.27)	0.31 (0.25 to 0.37)	0.19 (0.10 to 0.27)	0.17 (0.11 to 0.22)
**Cross-Trait Cross-Twin Correlations Between ADHD and Hypomania at Age 18 y**								
Age at ADHD symptom onset, y								
9 or 12	0.26 (0.21 to 0.30)		0.31 (0.27 to 0.35)	0.18 (0.13 to 0.24)	0.17 (0.10 to 0.23)	0.27 (0.22 to 0.32)	0.08 (0.02 to 0.15)	0.12 (0.07 to 0.17)
15	0.29 (0.24 to 0.34)		0.35 (0.31 to 0.40)	0.18 (0.11 to 0.25)	0.15 (0.06 to 0.22)	0.31 (0.25 to 0.37)	0.08 (0.00 to 0.15)	0.15 (0.09 to 0.22)
18	0.45 (0.41 to 0.48)		0.51 (0.48 to 0.54)	0.30 (0.23 to 0.35)	0.23 (0.15 to 0.30)	0.42 (0.37 to 0.46)	0.21 (0.13 to 0.28)	0.19 (0.14 to 0.24)
Age at hyperactivity-impulsivity onset, y								
9 or 12	0.28 (0.24 to 0.33)		0.32 (0.27 to 0.36)	0.23 (0.17 to 0.28)	0.15 (0.08 to 0.22)	0.29 (0.24 to 0.34)	0.09 (0.02 to 0.16)	0.12 (0.06 to 0.17)
18	0.48 (0.45 to 0.51)		0.52 (0.49 to 0.56)	0.32 (0.25 to 0.39)	0.18 (0.10 to 0.25)	0.41 (0.36 to 0.46)	0.20 (0.12 to 0.27)	0.16 (0.11 to 0.22)
Age at inattention onset, y								
9 or 12	0.22 (0.18 to 0.26)		0.25 (0.20 to 0.29)	0.14 (0.08 to 0.20)	0.15 (0.09 to 0.22)	0.20 (0.15 to 0.26)	0.06 (−0.01 to 0.12)	0.10 (0.05 to 0.15)
18	0.38 (0.34 to 0.42)		0.43 (0.40 to 0.47)	0.24 (0.18 to 0.30)	0.21 (0.14 to 0.28)	0.36 (0.31 to 0.41)	0.16 (0.09 to 0.23)	0.17 (0.12 to 0.22)

Abbreviations: ADHD, attention-deficit/hyperactivity disorder; DZF, dizygotic female twin pairs; DZM, dizygotic male twin pairs; DZOS, dizygotic opposite-sex twin pairs; MZF, monozygotic female twin pairs; MZM, monozygotic male twin pairs; NA, not applicable; *r*_ph,_ phenotypic correlation.

**Figure 2.**
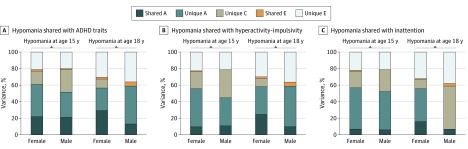
Proportion of the Genetic and Nonshared Environmental Risk Factors for Symptoms of Attention-Deficit/Hyperactivity Disorder (ADHD), Hyperactivity-Impulsivity, and Inattention Across Childhood and Adolescence That Are Associated With the Variation in Adolescent Hypomania by Sex

Phenotypic and cross-trait cross-twin correlations are presented in Table 3. An A, C, and E model was chosen as best fitting, with sibling interaction paths for ADHD. The proportions of variation in each hypomania scale that were associated with genetic and environmental risk factors unique to hypomania and shared with ADHD traits are shown in Figure 2. In total, 21% to 22% of the variance in hypomania at 15 years of age and 13% to 29% at 18 years of age was associated with genetic factors shared with ADHD at any age. Nonshared environmental factors associated with ADHD played a negligible role in hypomania. Hypomania-specific genetic factors accounted for 25% to 42% of its variance (eTable 5 in the Supplement). Similar results were found with the reduced hypomania scales (eTable 6 in the Supplement).

Hyperactivity-impulsivity was more strongly associated with hypomanic symptoms compared with inattention (Table 2). Up to 25% of the variance in hypomania was associated with genetic risk factors shared with hyperactivity-impulsivity and up to 16% with inattention (Figure 2). The models’ fit statistics and estimates are presented in eTables 7-9 in the Supplement. Analyses using the reduced hypomania scales yielded comparable findings (eTable 10 and eTable 11 in the Supplement).

## Discussion

To our knowledge, this was the first twin study to explore the shared genetic and environmental factors associated with ADHD and hypomania symptoms in youths. Traits of ADHD across childhood and adolescence were associated with adolescent hypomania. More than a quarter of the variance for hypomania was associated with shared genetic risk factors for ADHD traits (range, 13%-29%). Hypomania-specific genetic risk factors accounted for 27% to 46% of its variance. Environmental factors played a negligible role in the ADHD-hypomania symptom association. The genetic overlap with hypomania was larger with hyperactivity-impulsivity (range, 10%-25%) compared with inattention (range, 6%-16%).

The associations between ADHD and hypomanic symptoms observed here are similar to those reported in other adolescent samples.^2^ For example, 1 study^28^ found that among 98 adolescent patients with BD, 37.8% presented with ADHD, similar to the comorbidity rate of 37% in our study. Both ADHD dimensions were significantly associated with hypomania in this study, with a stronger association with hyperactivity-impulsivity. Previous research suggests that both ADHD domains are associated with BD to a similar degree^12^ or more strongly with inattention^10^ using data from adults^10^ and outpatients.^12^ We used a community sample of youths because the association between hypomania and the ADHD presentations may vary with age and from population to service level and assessment method.

This investigation provides a novel contribution by exploring the shared genetic and environmental factors associated with the ADHD-hypomania overlap in youths, examining hyperactivity-impulsivity and inattention separately. The percentage of variance in hypomania that could be associated with shared genetic factors with ADHD symptoms (range, 13%-29%) concurs with genetic correlations reported by a family study^14^ on BD II and ADHD (correlation, 0.33) and a molecular genetic study^29^ (correlation, 0.26).

Our other novel finding is that up to 25% of the variance in hypomania was associated with genetic factors related to hyperactivity-impulsivity compared with up to 16% for inattention.^30^ This finding is consistent with results showing that the degree to which ADHD symptom domains share genetic risk factors with other psychopathological dimensions in youths varies.^31^

Our findings have important clinical and research implications. First, our results provide additional evidence of the ADHD-hypomania symptom overlap in youths, highlighting the need for early identification and recognition among practitioners, especially given the care needed to avoid the negative outcomes associated with ADHD-BD comorbidity (eg, suicide attempts) compared with when these conditions occur alone.^11,12,13^ Future studies should follow up youths identified as high risk for BD and who exhibit ADHD symptoms to ascertain whether they develop specific forms of psychopathology. Second, significant genetic factors play a role in adolescent hypomania that are distinct from ADHD, suggesting that these phenotypes are not an extension of one another. Our results build on the ADHD and hypomania etiologic models, indicating shared genetic factors associated with these disorders. Uncovering the specific nature of the genetic overlap between these phenotypes should be the focus of further research.

### Strengths and Limitations

Strengths of this study include the use of a large, longitudinal, genetically informative sample; use of nationwide registries; and the distinction between the ADHD symptom domains in association with adolescent hypomanic traits; however, there are some limitations. First, hypomania was not measured in childhood; thus, the association between earlier hypomanic symptoms and ADHD traits across childhood and adolescence could not be assessed. Second, hypomanic and ADHD symptoms were measured using different instruments at each age, which may have affected the results. Both hypomania measures are considered among the best-validated and most discriminating adolescent BD instruments.^32^ The ADHD measures have good psychometric properties and are widely used.

Also, there has been much debate surrounding the overlap between ADHD and hypomania,^7^ particularly concerning their symptom similarity. In the current study, significant correlations between hypomania and ADHD symptoms were detected even when overlapping symptoms were removed from the hypomania scales, but various factors may still confound these associations. For instance, shared method variance may have inflated the associations because both phenotypes were measured using questionnaires.^33^

Several associated features of ADHD (eg, irritability and emotional liability) not included in its diagnostic criteria overlap with hypomania.^7^ These features were not accounted for here, which may have affected the ADHD-hypomania associations reported and should be considered in future research. Questionnaires are a practical data collection method for large samples needed to undertake twin research. However, they have various limitations, such as the reliance on a restricted number of items that sometimes lack context. Given that ADHD and hypomania were assessed using a restricted number of items, only 2 of 10 items for the CMRS and 5 of 13 for the Mood Disorders Questionnaire overlapped between these phenotypes and were removed to account for symptom similarity. It is possible that some overlapping symptoms were not measured.

Whether the presentation of overlapping symptoms is chronic or episodic is crucial to determining whether the symptoms are characteristic of ADHD or hypomania but was not clarified in all instruments used in this study (eg, CMRS). Standardized diagnostic interviews are a more comprehensive approach to establish the specific nature of the symptom presentation and should be adopted by future studies.

The ADHD-BD comorbidity was assessed using official diagnoses in the Swedish National Patient Register, but this method has several shortcomings that can inflate the comorbidity rate, although it was comparable to that in another study.^28^ These shortcomings include low agreement with standardized diagnostic interviews^34^ and questionable interrater reliability, particularly for BD.^35,36^ When BD is misdiagnosed, it is frequently misdiagnosed as ADHD,^37^ which affects the accuracy of the ADHD-BD comorbidity rate detected.

## Conclusions

To our knowledge, this was the first study to examine the shared genetic and environmental factors associated with ADHD traits across childhood and adolescence and adolescent hypomania in a representative, longitudinal twin cohort. The collective genetic risk factors for ADHD across childhood and adolescence may also be associated with hypomanic symptoms. The observed associations were stronger between hypomania and hyperactivity-impulsivity compared with inattention, but the association between inattention and hypomania was moderate and significant. This finding suggests that the overlap between ADHD and hypomania traits is likely to reflect a genetic link between these phenotypes. Nevertheless, a substantial amount of the variance for hypomania was associated with genetic risk factors that were not shared with ADHD.

## Notes

Supplement. **eTable 1.** Instrument Information **eTable 2.** Correlations Between ADHD Measures and Full and Reduced Hypomania Questionnaires **eTable 3.** Univariate Assumptions Testing **eTable 4.** Univariate Model Fit Statistics and Parameter Estimates **eTable 5.** Squared Standardized Path Coefficients From the Multivariate Cholesky Decomposition of ADHD Traits and Hypomania **eTable 6.** Squared Standardized Path Coefficients From the Multivariate Cholesky Decomposition of ADHD Traits and Reduced Hypomania Scales, Along With Estimates of Shared and Unique Environmental and Genetic Influences on Hypomania and ADHD **eTable 7.** Twin Model Fit Statistics **eTable 8.** Squared Path Coefficients From the Multivariate Cholesky Decomposition of Hyperactivity/Impulsivity and Hypomania **eTable 9.** Squared Path Coefficients From the Multivariate Cholesky Decomposition of Inattention and Hypomania **eTable 10.** Squared Standardized Path Coefficients From the Multivariate Cholesky Decomposition of Hyperactivity/Impulsivity and Reduced Hypomania Scales, Along With Estimates of Shared and Unique Environmental and Genetic Influences on Hypomania and Hyperactivity/Impulsivity **eTable 11.** Squared Standardized Path Coefficients From the Multivariate Cholesky Decomposition of Inattention and Reduced Hypomania Scales, Along With Estimates of Shared and Unique Environmental and Genetic Influences on Hypomania and Inattention **eMethods.** Analytical Code for Analysis
