# Supplementary material for: Association of Genetic and Environmental Risks for Attention-Deficit/Hyperactivity Disorder With Hypomanic Symptoms in Youths
Source: JAMA Psychiatry. 2019 Aug 14;76(11):1150–8. doi: 10.1001/jamapsychiatry.2019.1949 (PMC6694400; doi:10.1001/jamapsychiatry.2019.1949)
Supplement: Supplement. — eTable 1. Instrument Information eTable 2. Correlations Between ADHD Measures and Full and Reduced Hypomania Questionnaires eTable 3. Univariate Assumptions Testing eTable 4. Univariate Model Fit Statistics and Parameter Estimates eTable 5. Squared Standardized Path Coefficients From the Multivariate Cholesky Decomposition of ADHD Traits and Hypomania eTable 6. Squared Standardized Path Coefficients From the Multivariate Cholesky Decomposition of ADHD Traits and Reduced Hypomania Scales, Along With Estimates of Shared and Unique Environmental and Genetic Influences on Hypomania and ADHD eTable 7. Twin Model Fit Statistics eTable 8. Squared Path Coefficients From the Multivariate Cholesky Decomposition of Hyperactivity/Impulsivity and Hypomania eTable 9. Squared Path Coefficients From the Multivariate Cholesky Decomposition of Inattention and Hypomania eTable 10. Squared Standardized Path Coefficients From the Multivariate Cholesky Decomposition of Hyperactivity/Impulsivity and Reduced Hypomania Scales, Along With Estimates of Shared and Unique Environmental and Genetic Influences on Hypomania and Hyperactivity/Impulsivity eTable 11. Squared Standardized Path Coefficients From the Multivariate Cholesky Decomposition of Inattention and Reduced Hypomania Scales, Along With Estimates of Shared and Unique Environmental and Genetic Influences on Hypomania and Inattention eMethods. Analytical Code for Analysis [file jamapsychiatry-76-1150-s001.pdf]

Supplementary Online Content

Hosang GM, Lichtenstein P, Ronald A, Lundström S, Taylor MJ. Association of genetic and enviromental risks for attention-deficit/hyperactivity disorder with hypomanic symptoms in youths. *JAMA Psychiatry.* Published online August 14, 2019. doi:10.1001/jamapsychiatry.2019.1949

eTable 1. Instrument Information

eTable 2. Correlations Between ADHD Measures and Full and Reduced Hypomania Questionnaires

eTable 3. Univariate Assumptions Testing

eTable 4. Univariate Model Fit Statistics and Parameter Estimates

eTable 5. Squared Standardized Path Coefficients From the Multivariate Cholesky Decomposition of ADHD Traits and Hypomania

eTable 6. Squared Standardized Path Coefficients From the Multivariate Cholesky Decomposition of ADHD Traits and Reduced Hypomania Scales, Along With Estimates of Shared and Unique Environmental and Genetic Influences on Hypomania and ADHD

eTable 7. Twin Model Fit Statistics

eTable 8. Squared Path Coefficients From the Multivariate Cholesky Decomposition of Hyperactivity/Impulsivity and Hypomania

eTable 9. Squared Path Coefficients From the Multivariate Cholesky Decomposition of Inattention and Hypomania

eTable 10. Squared Standardized Path Coefficients From the Multivariate Cholesky Decomposition of Hyperactivity/Impulsivity and Reduced Hypomania Scales, Along With Estimates of Shared and Unique Environmental and Genetic Influences on Hypomania and Hyperactivity/Impulsivity

eTable 11. Squared Standardized Path Coefficients From the Multivariate Cholesky Decomposition of Inattention and Reduced Hypomania Scales, Along With Estimates of Shared and Unique Environmental and Genetic Influences on Hypomania and Inattention

eMethods. Analytical Code for Analysis

This supplementary material has been provided by the authors to give readers additional information about their work.

© 2019 Hosang GM et al. *JAMA Psychiatry.*

eTable 1. Instrument Information

Parent reports	Number of items	Score range	Cronbach's α	Rating scale	Validated cut-off score
**ADHD instruments**	-	-	-	-	-
A-TAC ADHD age 9/12	19	0-19	0.92	Yes/no	Broad cut-off: 6 or more<br>Strict cut-off: 12 or more
A-TAC hyperactivity-impulsivity subscale	10	0-10	0.87	Yes/no	
A-TAC inattention subscale	9	0-9	0.90	Yes/no	
Strengths and Difficulties Questionnaire, age 15[a]	5	0-8	0.75	3-point Likert scale	
Adult Behaviour Checklist [ABCL], age 18	13	0-24	0.83	3-point Likert scale	
ABCL hyperactivity-impulsivity subscale	6	0-12	0.75	3-point Likert scale	
ABCL inattention subscale	7	0-13	0.74	3-point Likert scale	
**Hypomania measures**	-	-	-	-	-
Child Mania Rating Scale, age 15	10	0-24	0.73	4-point Likert scale	10 or more
Mood Disorders Questionnaire, age 18	13	0-13	0.82	Yes/no	Minimum of 7 + symptoms must cluster in same period + score at least moderate impairment

Abbreviations: A-TAC, Autism-Tics, ADHD, and other Comorbidities Inventory; ABCL, Adult Behaviour Checklist

[a]Hyperactivity sub-scale

© 2019 Hosang GM et al. *JAMA Psychiatry.*

eTable 2. Correlations Between ADHD Measures and Full and Reduced Hypomania Questionnaires

	**Hypomania age 15**		**Hypomania age 18**	
	Full Scale	Reduced Scale [a]	Full Scale	Reduced Scale [b]
ADHD age 9/12	0.33 (0.27-0.38)	0.29 (0.24-0.35)	0.27 (0.19-0.33)	0.27 (0.20-0.34)
ADHD age 15	0.42 (0.37-0.47)	0.37 (0.32-0.42)	0.37 (0.27-0.45)	0.36 (0.27-0.45)
ADHD age 18	0.30 (0.20-0.39)	0.25 (0.15-0.35)	0.54 (0.49-0.60)	0.53 (0.47-0.58)
Hyperactivity age 9/12	0.32 (0.27-0.38)	0.30 (0.24-0.35)	0.24 (0.17-0.31)	0.24 (0.16-0.31)
Hyperactivity age 18	0.32 (0.23-0.41)	0.29 (0.20-0.39)	0.54 (0.48-0.59)	0.51 (0.46-0.57)
Inattention age 9/12	0.24 (0.18-0.30)	0.21 (0.15-0.27)	0.22 (0.14-0.29)	0.22 (0.15-0.29)
Inattention age 18	0.23 (0.13-0.32)	0.18 (0.08-0.28)	0.46 (0.39-0.51)	0.46 (0.49-0.52)

[a] Two items were removed from the Child Mania Rating Scale (item 5: Have periods of too much energy; item 7: talk so fast that he or she jumps from topic to topic)

[b] Five items were removed from the Mood Disorder Questionnaire (item 1: felt so good or hyper that other people thought they were not their normal self. Item 5: much more talkative or spoke faster than usual. Item 7: so easily distracted by things around them that they had trouble concentrating. Item 8: had much more energy than usual. Item 9: much more active or did many more things than usual.

© 2019 Hosang GM et al. *JAMA Psychiatry.*

eTable 3. Univariate Assumptions Testing

Model	-2LL	Parameters	df	AIC	BIC	$\Delta\chi^2$	Δdf	p
**ADHD Age 9/12**								
Fully Saturated	72345.47	26	26995	18355.47	-184452.91	-----	-----	-----
Submodel 1	72354.57	22	26999	18356.57	-184481.86	9.10	4	.06
Submodel 2	72358.48	18	27003	18352.48	-184516.00	13.01	8	.11
Submodel 3	72391.13	16	27005	18381.13	-184502.37	45.66	10	<.001
Submodel 4	72416.61	14	27007	18402.61	-184495.91	71.14	12	<.001
**ADHD Age 15**								
Fully Saturated	26356.45	26	9557	7242.45	-64557.50	-----	-----	-----
Submodel 1	26357.22	22	9561	7235.22	-64594.78	0.78	4	.94
Submodel 2	26359.57	18	9565	7229.57	-64630.49	3.12	8	.93
Submodel 3	26373.61	16	9567	7239.61	-64635.47	17.16	10	.07
Submodel 4	26381.41	14	9569	7243.41	-64646.69	24.97	12	<.05
**ADHD Age 18**								
Fully Saturated	11953.89	26	4319	3315.89	-29131.95	-----	-----	-----
Submodel 1	11954.86	22	4323	3308.86	-29169.02	0.98	4	.91
Submodel 2	11959.68	18	4327	3305.68	-29202.26	5.79	8	.67
Submodel 3	11965.18	16	4329	3307.18	-29215.79	11.29	10	.34
Submodel 4	11970.96	14	4331	3308.96	-29229.03	17.07	12	.15
**Hyperactivity/Impulsivity Age 9/12**								
Fully Saturated	72901.13	26	27012	18877.13	-184058.96	-----	-----	-----
Submodel 1	72907.43	22	27016	18875.43	-184090.71	6.30	4	.18
Submodel 2	72912.67	18	27020	18872.67	-184123.52	11.54	8	.17
Submodel 3	72933.02	16	27022	18889.02	-184122.20	31.88	10	<.001
Submodel 4	72976.12	14	27024	18928.12	-184098.13	74.99	12	<.001

© 2019 Hosang GM et al. *JAMA Psychiatry.*

**Inattention Age 9/12**								
Fully Saturated	73365.51	26	27008	19349.51	-183556.53	-----	-----	-----
Submodel 1	73372.66	22	27012	19348.66	-183587.43	7.15	4	.13
Submodel 2	73377.19	18	27016	19345.19	-183620.95	11.68	8	.17
Submodel 3	73412.490	16	27018	19376.49	-183604.68	46.98	10	<.001
Submodel 4	73448.47	14	27020	19408.47	-183587.73	82.96	12	<.001
**Hyperactivity/Impulsivity Age 18**								
Fully Saturated	12163.22	26	4332	3499.22	-29046.28	-----	-----	-----
Submodel 1	12165.92	22	4336	3493.92	-29081.63	2.70	4	.61
Submodel 2	12172.87	18	4340	3492.87	-29112.73	9.65	8	.29
Submodel 3	12182.83	16	4342	3498.83	-29121.81	19.61	10	<.05
Submodel 4	12199.75	14	4344	3511.75	-29123.91	36.53	12	<.001
**Inattention Age 18**								
Fully Saturated	11998.99	26	4332	3334.99	-29210.51	-----	-----	-----
Submodel 1	12001.23	22	4336	3329.23	-29246.32	2.24	4	.69
Submodel 2	12003.85	18	4340	3323.85	-29281.75	4.86	8	.77
Submodel 3	12006.78	16	4342	3322.78	-29297.85	7.78	10	.65
Submodel 4	12011.63	14	4344	3323.63	-29312.03	12.64	12	.40
**Hypomania Age 15**								
Fully Saturated	16351.90	26	6330	3691.90	-43864.20	-----	-----	-----
Submodel 1	16359.23	22	6334	3691.23	-43894.92	7.33	4	.12
Submodel 2	16361.67	18	6338	3685.67	-43930.54	9.77	8	.28
Submodel 3	16362.97	16	6340	3682.97	-43948.26	11.07	10	.36
Submodel 4	16366.17	14	6342	3682.17	-43964.09	14.27	12	.28
**Hypomania Age 18**								
Fully Saturated	11934.89	26	4374	3186.89	-29674.15	-----	-----	-----
Submodel 1	11937.35	22	4378	3181.34	-29709.75	2.45	4	.65
Submodel 2	11943.00	18	4382	3179.00	-29742.15	8.11	8	.42
Submodel 3	11944.93	16	4384	3176.93	-29759.24	10.03	10	.44
Submodel 4	11947.94	14	4386	3175.94	-29775.25	13.05	12	.37

The models detailed in this table test a number of assumptions of the twin method. These include the following: 1) that means and variances are equal within twin pairs; 2) that means and variances are equal across different zygosity groups. These assumptions are thus tested by first fitting the fully saturated model (which allows means and variances to differ across sex and zygosity groups, and within twin pair. The following submodels were then fitted and compared to the fully saturated models: submodel 1) equates means within twin pairs in same-sex twins to test the assumption of equal means within twin pairs; submodel 2) additionally equates variances within twin pairs in same-sex twins to test the assumption of equal variances within twin pairs; submodel 3) additionally equates the means across same-sex zygosity groups (but still allowing the means to differ by sex) to test the assumption of equal means across zygosity; and submodel 4) additionally equates variances across same-sex zygosity groups (but still allowing variances to differ by sex) to test the assumption of equal variances across zygosity.

-2LL: fit statistic that is -2 times the log-likelihood of the data; df: degrees of freedom; BIC: Bayesian Information Criteria; $\Delta\chi^2$: difference in -2LL between each submodel and the fully saturated model, which is χ^2 distributed; Δdf: change in degrees of freedom between two models.

© 2019 Hosang GM et al. *JAMA Psychiatry.*

eTable 4. Univariate Model Fit Statistics and Parameter Estimates

**ADHD Age 9/12**								
Model	-2LL	Parameters	df	AIC	BIC	$\Delta\chi^2$	Δdf	p
Saturated	72345.47	26	26995	18355.47	-184452.91	-----	-----	-----
ADE-s [a]	72452.34	12	27009	18434.34	-184479.21	106.87	14	<.001
ACE [a]	72507.25	9	27012	18483.25	-184452.84	161.78	17	<.001
ADE-s H [b]	72701.89	7	27014	18673.89	-184277.23	249.55	5	<.001
ADE [b]	72476.92	9	27012	18452.92	-184483.18	24.57	3	<.001
AE-s [b]	***72452.34***	***10***	***27011***	***18430.34***	***-184498.24***	***0.00***	***2***	***1.00***
AE [b]	72507.25	7	27014	18479 .25	-184471.87	54.91	5	<.001
E [b]	75373.67	5	27016	21341.67	-181624.47	2921.33	7	<.001

	A	D	C	E	s
Male	.74 (.72-.77)	-----	-----	.26 (.23-.28)	-.07 (-.09/-.03)
Female	.69 (.65-.72)	-----	-----	.31 (.28-.35)	-.05 (-.08/-.03)

**ADHD Age 15**								
Model	-2LL	Parameters	df	AIC	BIC	$\Delta\chi^2$	Δdf	p
Saturated	26356.45	26	9557	7242.45	-64557.50	-----	-----	-----
ADE-s [a]	26391.26	12	9571	7249.26	-64655.87	34.82	14	<.01
ADE-s H [b]	26399.05	7	9576	7247.05	-64695.65	7.79	5	.17
ADE [c]	26406.08	6	9577	7252.07	-64698.13	7.03	1	<.01
AE-s [c]	***26399.27***	***6***	***9577***	***7245.27***	***-64704.93***	***0.22***	***1***	***.64***
AE [c]	26482.92	5	9578	7326.92	-64630.80	83.87	2	<.001
E [c]	26964.85	4	9579	7806.85	-64158.39	565.80	3	<.001

	A	D	C	E	s
Estimate	.68 (.64-.72)	-----	-----	.32 (.28-.36)	-.12 (-.14/-.09)

© 2019 Hosang GM et al. *JAMA Psychiatry.*

ADHD Age 18

Model	-2LL	Parameters	df	AIC	BIC	$\Delta\chi^2$	Δdf	p
Saturated	11953.89	26	4319	3315.89	-29131.95	-----	-----	-----
ADE [a]	11982.53	9	4336	3310.53	-29265.03	28.64	17	<.05
ADE-H [b]	11987.85	6	4339	3309.85	-29288.24	5.33	3	.15
AE [c]	***11991.54***	***5***	***4340***	***3311.54***	***-29294.07***	***3.67***	***1***	***.05***
E [c]	12265.50	4	4341	3583.50	-29029.62	277.65	2	<.001

	A	D	C	E	s
Estimate	.51 (.46-.55)	-----	-----	.49 (.45-.54)	-----

Hyperactivity/Impulsivity Age 9/12

Model	-2LL	Parameters	df	AIC	BIC	$\Delta\chi^2$	Δdf	p
Saturated	72901.13	26	27012	18877.13	-184058.96	-----	-----	-----
ADE-s [a]	72976.14	12	27026	18924.14	-184117.13	75.01	14	<.001
ACE [a]	73150.54	9	27029	19092.54	-183971.27	249.40	17	<.001
ADE-s H [b]	73268.19	7	27031	19206.19	-183872.65	292.05	5	<.001
ADE [b]	73004.47	9	27029	18946.47	-184117.34	28.33	3	<.001
AE-s [b]	72976.14	10	27028	18920.14	-184136.16	0.00	2	1.00
AE [b]	73152.40	7	27031	19090.40	-183988.43	176.26	5	<.001
E [b]	75604.75	5	27033	21538.75	-181555.12	2628.61	7	<.001

	A	D	C	E	s
Male	.77 (.74-.79)	-----	-----	.23 (.21-.26)	-.10 (-.13/-.08)
Female	.73 (.71-.76)	-----	-----	.27 (.24-.29)	-.10 (-.12/-.07)

© 2019 Hosang GM et al. *JAMA Psychiatry.*

Inattention Age 9/12

Model	-2LL	Parameters	df	AIC	BIC	Δχ²	Δdf	p
Saturated	73365.51	26	27008	19349.51	-183556.53	-----	-----	-----
ADE-s [a]	73496.34	12	27022	19452.34	-183558.88	130.83	14	<.001
ACE [a]	73668.63	9	27025	19618.63	-183415.12	303.13	17	<.001
ADE-s H [b]	73882.87	7	27027	19828.87	-183219.92	386.53	5	<.001
ADE [b]	73531.94	9	27025	19481.94	-193551.82	35.61	3	<.001
AE-s [b]	73496.34	10	27024	19448.34	-183577.91	0.00	2	1.00
AE [b]	73668.67	7	27027	19614.67	-183434.11	172.33	5	<.001
E [b]	75196.38	5	27029	21138.38	-181925.43	1700.04	7	<.001

	A	D	C	E	s
Male	.72 (.69-.75)	-----	-----	.28 (.25-.31)	-.12 (-.14/-.10)
Female	.66 (.62-.69)	-----	-----	34 (.31-.38)	-.11 (-.13/-.08)

Hyperactivity/Impulsivity Age 18

Model	-2LL	Parameters	df	AIC	BIC	Δχ²	Δdf	p
Saturated	12163.22	26	4332	3499.22	-29046.28	-----	-----	-----
ADE-s [a]	12192.63	12	4346	3500.63	-29150.06	29.40	14	<.01
ACE [a]	12217.17	9	4349	3519.17	-29154.06	53.94	17	<.001
ADE-s H [b]	12199.64	7	4351	3497.64	-29190.61	7.01	5	.22
ADE [c]	12208.12	6	4352	3504.12	-29191.64	8.49	1	<.01
AE-s [c]	12199.64	6	4352	3495.64	-29200.12	0.00	6	1.00
AE [c]	12222.37	5	4353	3516.36	-29186.91	22.72	2	<.001
E [c]	12352.66	4	4354	3644.66	-29066.13	153.02	3	<.001

	A	D	C	E	s
	.60 (.52-.67)	-----	-----	.40 (.33-.48)	-.11 (-.15/-.07)

© 2019 Hosang GM et al. *JAMA Psychiatry.*

Inattention Age 18

Model	-2LL	Parameters	df	AIC	BIC	$\Delta\chi^2$	Δdf	p
Saturated	11998.99	26	4332	3334.99	-29210.51	-----	-----	-----
ADE [a]	12034.24	9	4339	3336.24	-29336.98	35.25	17	<.01
ADE H [b]	12049.28	6	4352	3345.28	-29350.48	15.04	3	<.01
AE [b]	12046.33	7	4351	3344.33	-29343.92	12.09	2	<.01
E [b]	12254.17	5	4353	3548.17	-29155.10	219.93	4	<.001

	A	D	C	E	s
Male	.24 (.00-.53)	.29 (.00-.58)	-----	.47 (.40-.55)	-----
Female	.08 (.00-.28)	.39 (.17-.52)	-----	.53 (.46-.61)	-----

Hypomania Age 15

Model	-2LL	Parameters	df	AIC	BIC	$\Delta\chi^2$	Δdf	p
Saturated	16351.90	26	6330	3691.90	-43864.20	-----	-----	-----
ACE [a]	16368.84	9	6347	3674.84	-44008.98	16.94	17	.46
ACE H [b]	***16374.29***	***6***	***6350***	***3674.29***	***-44032.07***	***5.45***	***3***	***.14***
AE [c]	16440.20	5	6351	3738.20	-43975.67	65.92	1	<.001
CE [c]	16552.55	5	6351	3850.55	-43863.32	178.26	1	<.001
E [c]	17958.56	4	6352	5254.56	-42466.82	1584.27	2	<.001

	A	D	C	E	s
Estimate	.50 (.44-.57)	-----	.27 (.21-33)	.22 (.20-.25)	-----

© 2019 Hosang GM et al. *JAMA Psychiatry.*

Hypomania Age 18								
Model	-2LL	Parameters	df	AIC	BIC	Δχ²	Δdf	p
Saturated	11934.89	26	4374	3186.89	-29674.15	-----	-----	-----
ADE [a]	11956.36	9	4391	3174.36	-29814.40	21.47	17	.21
ACE [a]	11952.66	9	4391	3170.66	-29818.10	17.77	17	.40
ACE H [b]	11959.32	6	4394	3171.32	-29839.98	6.66	3	.08
AE [c]	***11959.47***	***5***	***4395***	***3169.47***	***-29849.34***	***0.15***	***1***	***.70***
CE [c]	12047.92	5	4395	3257.92	-29760.89	88.60	1	<.001
E [c]	12453.97	4	4396	3661.97	-29364.35	494.65	2	<.001

	A	D	C	E	s
	.64 (.60-.67)	-----	-----	.36 (.33-.40)	-----

The best-fitting model for each variable is highlighted in bold italics.

-2LL: fit statistic that is -2 times the log-likelihood of the data; df: degrees of freedom; BIC: Bayesian Information Criteria; $\Delta\chi^2$: difference in -2LL between two models, distributed χ^2; Δdf difference in degrees of freedom between two models

A: additive genetic influences; D: non-additive genetic influences; C: shared environmental influences; E: nonshared environmental influences; s: sibling interaction effects; Q: quantitative sex limitation; H: homogeneity (i.e. no sex differences)

[a] These models are compared to the saturated model

[b] The homogeneity models are compared to the full ADE-s, ADE, or ACE model; in the event that a homogeneity model does not fit the data, the AE-s, ADE, AE, CE, and E models in the lower parts of the tables are also compared to the full ADE-s, ADE, or ACE model

[c] In these instances, the homogeneity model was not a significantly poorer fit than the model with quantitative sex limitation and so the AE-s, ADE, AE, CE, and E models shown in the lower part of tables are compared to the homogeneity model

© 2019 Hosang GM et al. *JAMA Psychiatry.*

eTable 5. Squared Standardized Path Coefficients From the Multivariate Cholesky Decomposition of ADHD Traits and Hypomania

		A					C		E				
		A1	A2	A3	A4	A5	C3	C5	E1	E2	E3	E4	E5
ADHD age 9/12	Females	.68 (.66-.71)							.32 (.29-.36)				
	Males	.74 (.71-.76)							.26 (.24-.29)				
ADHD age 15	Females	.16 (.13-.19)	.54 (.50-.59)						.02 (.01-.04)	.27 (.23-.32)			
	Males	.20 (.17-.23)	.48 (.42-.53)						.02 (.01-.04)	.30 (.26-.36)			
Hypomania age 15	Females	.10 (.07-.13)	.12 (.09-.16)	.39 (.30-.46)			.16 (.11-.23)		.01 (.00-.02)	.01 (.00-.02)	.21 (.18-.23)		
	Males	.09 (.07-.12)	.12 (.09-.15)	.30 (.21-.40)			.27 (.19-.35)		.00 (.00-.01)	.01 (.00-.02)	.20 (.18-.23)		
ADHD age 18	Females	.19 (.14-.24)	.09 (.06-.13)	.04 (.01-.08)	.30 (.21-.38)				.02 (.02-.04)	.02 (.01-.04)	.00 (.00-.01)	.34 (.28-.43)	
	Males	.12 (.08-.16)	.11 (.07-.16)	.03 (.01-.08)	.31 (.20-.40)				.07 (.04-.12)	.03 (.01-.06)	.01 (.00-.02)	.32 (.24-.42)	
Hypomania age 18	Females	.11 (.08-.15)	.07 (.03-.11)	.02 (.00-.06)	.11 (.06-.16)	.25 (.11-.39)		.11 (.00-.23)	.01 (.00-.02)	.00 (.00-.01)	.01 (.00-.03)	.01 (.01-.03)	.29 (.25-.34)
	Males	.05 (.03-.08)	.02 (.00-.05)	.07 (.02-.16)	.06 (.02-.12)	.39 (.30-.46)		.00 (.00-.04)	.01 (.00-.03)	.02 (.00-.04)	.01 (.00-.04)	.02 (.00-.04)	.35 (.30-.42)

Each estimate represents the proportion of variation in each measure accounted for by each variance component.

A1-A5 represent the additive genetic variance components; A1: additive genetic influences on ADHD traits at age 9/12, which also influence all four other phenotypes in the model; A2: additive genetic influences on ADHD traits at age 15; A3: additive genetic influences on ADHD traits at age 18; A4: additive genetic influences on hypomania at age 15; A5: additive genetic influences on hypomania at age 18

E1-E5 are the equivalent pathways for nonshared environmental variance components; C4 and C5 are the shared environmental pathways influencing hypomania, which only influence hypomania and not ADHD traits

© 2019 Hosang GM et al. *JAMA Psychiatry.*

eTable 6. Squared Standardized Path Coefficients From the Multivariate Cholesky Decomposition of ADHD Traits and Reduced Hypomania Scale, Along With Estimates of Shared and Unique Environmental and Genetic Influences on Hypomania and ADHD

		A					C		E				
		A1	A2	A3	A4	A5	C3	C5	E1	E2	E3	E4	E5
ADHD age 9/12	Females	.74 (.71-.76)							.26 (.24-.29)				
	Males	.77 (.75-.79)							.23 (.21-.25)				
ADHD age 15	Females	.22 (.19-.25)	.52 (.48-.56)						.02 (.01-.03)	.24 (.21-.28)			
	Males	.27 (.24-.31)	.46 (.41-.50)						.02 (.01-.04)	.24 (.21-.29)			
Hypomania age 15	Females	.11 (.08-.14)	.12 (.09-.15)	.42 (.35-.49)			.11 (.06-.18)		.02 (.01-.03)	.01 (.01-.02)	.20 (.18-.23)		
	Males	.10 (.07-.13)	.10 (.08-.14)	.46 (.37-.54)			.16 (.09-.24)		.00 (.00-.01)	.00 (.00-.01)	.17 (.15-.19)		
ADHD age 18	Females	.28 (.23-.33)	.13 (.09-.18)	.02 (.01-.05)	.34 (.28-.40)				.00 (.00-.01)	.01 (.00-.02)	.00 (.00-.01)	.21 (.17-.26)	
	Males	.20 (.15-.24)	.12 (.08-.17)	.01 (.00-.03)	.40 (.33-.45)				.07 (.04-.11)	.03 (.01-.06)	.01 (.00-.02)	.17 (.13-.22)	
Hypomania age 18	Females	.13 (.09-.18)	.06 (.03-.10)	.02 (.00-.05)	.09 (.06-.14)	.25 (.13-.33)		.05 (.00-.14)	.01 (.00-.03)	.01 (.00-.02)	.02 (.00-.05)	.04 (.02-.07)	.33 (.28-.38)
	Males	.04 (.02-.08)	.03 (.01-.07)	.05 (.02-.12)	.07 (.03-.11)	.35 (.27-.42)		.00 (.00-.04)	.04 (.01-.08)	.02 (.00-.05)	.01 (.00-.04)	.06 (.03-.10)	.34 (.28-.41)

Proportion of the variance of adolescent hypomania (using the reduced scales) that are shared with ADHD symptoms in childhood and adolescence

	Hypomania age 15					Hypomania age 18				
	Shared A	Unique A	Unique C	Shared E	Unique E	Shared A	Unique A	Unique C	Shared E	Unique E
Females	24%	42%	11%	3%	20%	28%	27%	5%	6%	35%
Males	20%	46%	16%	0%	17%	14%	40%	0%	12%	35%

© 2019 Hosang GM et al. *JAMA Psychiatry.*

eTable 7. Twin Model Fit Statistics

Model	-2LL	Parameters	df	BIC	$\Delta\chi^2$	Δdf	p
**ADHD traits and hypomania**							
Model	-2LL	Parameters	df	BIC	$\Delta\chi^2$	Δdf	p
Saturated	133541.40	330	51375	-355179.34	-----	-----	-----
ACE, Sex Lim [a]	134039.26	105	51600	-356821.87	497.86	225	<.001
ADE-s, Sex Lim [a]	133974.20	114	51591	-356801.31	432.8	216	<.001
ADCE-s, Sex Lim [a]	133939.30	94	51611	-357026.46	397.9	236	<.001
ADCE-s, Hom. [b]	134255.12	56	51649	-357072.13	315.82	38	<.001
ACE-s, Sex Lim [b]	***133939.30***	***88***	***51617***	***-357083.54***	***0.00***	***6***	***1***
ADE-s, Sex Lim [b]	134003.64	90	51615	-357000.18	64.34	4	<.001
AE-s, Sex Lim [b]	134003.64	84	51621	-357057.25	64.34	10	<.001
AE, Sex Lim [b]	134139.79	75	51630	-357006.72	200.49	19	<.001
E, Sex Lim [b]	139457.55	45	51660	-351974.34	5518.25	49	<.001
**Hyperactivity-impulsivity and hypomania**							
Model	-2LL	Parameters	df	BIC	$\Delta\chi^2$	Δdf	p
Saturated	110800.02	224	41928	-288053.19	-----	-----	-----
ACE, Sex Lim [a]	111226.16	72	42080	-289072.99	426.15	152	<.001
ADE-s, Sex Lim [a]	111126.11	78	42074	-289115.97	326.09	146	<.001
ADCE-s, Sex Lim [a]	111053.57	66	42086	-289302.66	253.55	158	<.001
ADCE-s, Hom. [b]	112639.85	40	42112	-287963.71	1586.28	26	<.001
ACE-s, Sex Lim [b]	***111057.27***	***62***	***42090***	***-289337.01***	***3.70***	***4***	***0.45***
ADE-s, Sex Lim [b]	111132.49	62	42090	-289261.79	78.92	4	<.001
AE-s, Sex Lim [b]	111137.30	58	42094	-289295.03	83.74	8	<.001
AE, Sex Lim [b]	111341.16	52	42100	-289148.25	287.59	14	<.001
E, Sex Lim [b]	115782.79	32	42120	-284896.88	4729.22	34	<.001
**Inattention and hypomania**							
Model	-2LL	Parameters	df	BIC	$\Delta\chi^2$	Δdf	p
Saturated	111638.00	224	41924	-287177.16	-----	-----	-----
ACE, Sex Lim [a]	112110.59	72	42076	-288150.51	472.59	152	<.001
ADE-s, Sex Lim [a]	111980.71	78	42070	-288223.31	342.71	146	<.001
ADCE-s, Sex Lim [a]	111937.37	66	42082	-288380.81	299.38	158	<.001
ADCE-s, Hom [b]	113542.12	40	42108	-287023.39	1604.75	26	<.001
ACE-s, Sex Lim [b]	***111937.37***	***62***	***42086***	***-288418.86***	***0.00***	***4***	***1.00***
ADE-s, Sex Lim [b]	111996.73	62	42086	-288359.5	59.36	4	<.001
AE-s, Sex Lim [b]	111996.73	58	42090	-288397.55	59.36	8	<.001
AE, Sex Lim [b]	112186.41	52	42096	-288264.94	249.04	14	<.001
E, Sex Lim [b]	115799.65	32	42116	-284841.96	3862.28	34	<.001

Bold italics denote the best fitting model according to BIC

Sex Lim: model with quantitative sex limitation, which allowed each parameter to differ by sex

ACE, Hom: homogeneity model, which constrains all variance components to be equal by sex

-2LL: fit statistic that is -2 times the log-likelihood of the data; df: degrees of freedom; BIC: Bayesian Information Criteria; $\Delta\chi^2$: difference in -2LL between two models, which is χ^2 distributed; Δdf: difference in degrees of freedom between two models

[a] *The comparative fit statistics (i.e. $\Delta\chi^2$, Δdf, and p-values) are comparisons with the saturated model*

[b] *The comparative fit statistics (i.e. $\Delta\chi^2$, Δdf, and p-values) are comparisons with the ADCE-s sex limitation model*

© 2019 Hosang GM et al. *JAMA Psychiatry.*

eTable 8. Squared Path Coefficients From the Multivariate Cholesky Decomposition of Hyperactivity/Impulsivity and Hypomania

		A				C		E			
		A1	A2	A3	A4	C2	C4	E1	E2	E3	E4
Hyperactivity-Impulsivity age 9/12	Females	.73 (.72-.75)						.27 (.25-.30)			
	Males	.77 (.75-.79)						.23 (.22-.25)			
Hypomania age 15	Females	.10 (.07-.13)	.47 (.39-.55)			.21 (.14-.28)		.01 (.00-.01)	.22 (.19-.25)		
	Males	.11 (.08-.14)	.34 (.24-.45)			.34 (.24-.43)		.00 (.00-.01)	.21 (.19-.25)		
Hyperactivity-Impulsivity age 18	Females	.17 (.13-.22)	.09 (.05-.15)	.42 (.35-.48)				.01 (.00-.02)	.01 (.00-.02)	.30 (.26-.35)	
	Males	.14 (.10-.18)	.15 (.07-.27)	.25 (.13-.35)				.03 (.01-.06)	.01 (.00-.03)	.42 (.37-.56)	
Hypomania age 18	Females	.13 (.09-.17)	.08 (.04-.13)	.12 (.07-.17)	.26 (.12-.39)		.10 (.00-.22)	.00 (.00-.01)	.01 (.00-.03)	.02 (.01-.03)	.29 (.25-.34)
	Males	.07 (.04-.11)	.14 (.06-.26)	.03 (.00-.10)	.35 (.24-.42)		.00 (.00-.04)	.01 (.00-.03)	.00 (.00-.03)	.04 (.02-.06)	.36 (.31-.42)

Each estimate represents the proportion of variation in each measure accounted for by each variance component.

A1-A4 represent the additive genetic variance components; A1: additive genetic influences on hyperactivity/impulsivity at age 9/12, which also influence all four other phenotypes in the model; A2: additive genetic influences on hyperactivity/impulsivity at age 18; A3: additive genetic influences on hypomania at age 15; A4: additive genetic influences on hypomania at age 18

E1-E4 are the equivalent pathways for nonshared environmental variance components; C3-C4 are the shared environmental influences on hypomania, which were not estimated for hyperactivity/impulsivity and do not contribute to the phenotypic covariance in the model.

Sibling interaction pathway estimates for hyperactivity/impulsivity at age 9/12: female=-.09 (-.10/-.07), male=-.10 (-.13/-.08), opposite-sex=-.07 (-.09/-.08); sibling interaction pathway estimates for hyperactivity/impulsivity at age 18: female=-.14 (-.19/-.10), male=-.07 (-.09/-.05), opposite-sex=-.10 (-.12/-.08)

© 2019 Hosang GM et al. *JAMA Psychiatry.*

eTable 9. Squared Path Coefficients From the Multivariate Cholesky Decomposition of Inattention and Hypomania

		A				C		E			
		A1	A2	A3	A4	C2	C4	E1	E2	E3	E4
Inattention age 9/12	Male	.72 (.69-.75)						.28 (.25-.31)			
	Female	.66 (.62-.69)						.34 (.31-.38)			
Hypomania age 15	Male	.06 (.04-.09)	.46 (.36-.57)			.26 (.17-.35)		.00 (.00-.01)	.21 (.19-.24)		
	Female	.07 (.05-.10)	.50 (.40-.58)			.20 (.13-.29)		.01 (.00-.01)	.22 (.19-.25)		
Inattention age 18	Male	.11 (.07-.16)	.09 (.04-.16)	.40 (.27-.49)				.07 (.04-.12)	.00 (.00-.02)	.32 (.25-.43)	
	Female	.15 (.10-.19)	.09 (.05-.16)	.40 (.29-.49)				.02 (.00-.05)	.00 (.00-.02)	.33 (.26-.43)	
Hypomania age 18	Male	.04 (.02-.07)	.10 (.05-.19)	.03 (.01-.08)	.42 (.34-.49)		.00 (.00-.04)	.01 (.00-.03)	.01 (.00-.04)	.02 (.00-.04)	.37 (.31-.43)
	Female	.07 (.04-.11)	.10 (.05-.16)	.09 (.04-.14)	.30 (.15-.43)		.11 (.00-.24)	.00 (.00-.02)	.01 (.00-.03)	.01 (.00-.02)	.31 (.27-.36)

Each estimate represents the proportion of variation in each measure accounted for by each variance component.

A1-A4 represent the additive genetic variance components; A1: additive genetic influences on inattention at age 9/12, which also influence all four other phenotypes in the model; A2: additive genetic influences on inattention at age 18; A3: additive genetic influences on hypomania at age 15; A4: additive genetic influences on hypomania at age 18

E1-E4 are the equivalent pathways for nonshared environmental variance components

© 2019 Hosang GM et al. *JAMA Psychiatry.*

eTable 10. Squared Standardized Path Coefficients From the Multivariate Cholesky Decomposition of Hyperactivity/Impulsivity and Reduced Hypomania Scale, Along With Estimates of Shared and Unique Environmental and Genetic Influences on Hypomania and Hyperactivity/Impulsivity

		A				C		E			
		A1	A2	A3	A4	C2	C4	E1	E2	E3	E4
Hyperactivity-Impulsivity age 9/12	Females	.77 (.75-.79)						.23 (.21-.25)			
	Males	.79 (.77-.81)						.21 (.19-.23)			
Hypomania age 15	Females	.09 (.07-.12)	.52 (.43-.60)			.15 (.08-.23)		.02 (.01-.03)	.22 (.19-.25)		
	Males	.11 (.08-.14)	.54 (.44-.62)			.18 (.10-.26)		.00 (.00-.01)	.17 (.15-.20)		
Hyperactivity-Impulsivity age 18	Females	.22 (.17-.27)	.11 (.06-.17)	.48 (.41-.55)				.00 (.00-.01)	.00 (.00-.01)	.19 (.15-.23)	
	Males	.20 (.15-.26)	.08 (.03-.14)	.42 (.31-.50)				.03 (.01-.06)	.01 (.00-.04)	.26 (.19-.35)	
Hypomania age 18	Females	.13 (.09-.18)	.05 (.02-.11)	.10 (.06-.15)	.29 (.18-.36)		.02 (.00-.11)	.01 (.00-.02)	.02 (.00-.05)	.04 (.02-.06)	.34 (.29-.40)
	Males	.06 (.03-.10)	.09 (.04-.17)	.06 (.02-.11)	.32 (.23-.40)		.01 (.00-.07)	.02 (.00-.05)	.00 (.00-.04)	.07 (.04-.12)	.36 (.30-.43)

Proportion of the variance of adolescent hypomania (using the reduced scales) that are shared with hyperactivity/impulsivity

	Hypomania age 15					Hypomania age 18				
	Shared A	Unique A	Unique C	Shared E	Unique E	Shared A	Unique A	Unique C	Shared E	Unique E
Females	9%	52%	15%	2%	22%	23%	34%	2%	5%	36%
Males	11%	54%	18%	0%	17%	12%	41%	1%	9%	36%

© 2019 Hosang GM et al. *JAMA Psychiatry.*

eTable 11. Squared Standardized Path Coefficients From the Multivariate Cholesky Decomposition of Inattention and Reduced Hypomania Scale, Along With Estimates of Shared and Unique Environmental and Genetic Influences on Hypomania and Inattention

		A				C		E			
		A1	A2	A3	A4	C2	C4	E1	E2	E3	E4
Inattention age 9/12	Females	.68 (.65-.71)						.32 (.29-.35)			
	Males	.75 (.73-.77)						.25 (.23-.27)			
Hypomania age 15	Females	.07 (.05-.11)	.55 (.45-.64)			.14 (.06-.22)		.01 (.00-.02)	.23 (.20-.26)		
	Males	.05 (.03-.08)	.61 (.52-.70)			.15 (.08-.24)		.00 (.00-.01)	.18 (.15-.21)		
Inattention 18	Females	.17 (.13-.22)	.09 (.04-.15)	.43 (.34-.51)				.01 (.00-.03)	.00 (.00-.01)	.29 (.23-.37)	
	Males	.15 (.11-.20)	.05 (.02-.09)	.51 (.43-.57)				.07 (.04-.12)	.00 (.00-.02)	.21 (.17-.28)	
Hypomania age 18	Females	.08 (.05-.12)	.08 (.03-.14)	.09 (.06-.15)	.27 (.13-.38)		.07 (.00-.18)	.01 (.00-.02)	.03 (.00-.06)	.03 (.01-.05)	.36 (.30-.42)
	Males	.02 (.01-.05)	.09 (.04-.16)	.05 (.02-.08)	.38 (.29-.46)		.00 (.00-.04)	.03 (.01-.07)	.01 (.00-.04)	.04 (.02-.08)	.37 (.31-.45)

Proportion of the variance of adolescent hypomania (using the reduced scales) that are shared with inattention

	Hypomania age 15					Hypomania age 18				
	Shared A	Unique A	Unique C	Shared E	Unique E	Shared A	Unique A	Unique C	Shared E	Unique E
Females	7%	55%	14%	1%	23%	17%	55%	7%	1%	23%
Males	5%	61%	15%	0%	18%	7%	47%	0%	7%	38%

© 2019 Hosang GM et al. *JAMA Psychiatry.*

eMethods. Analytic Code for Analyses

```
#-----
# Program: multivariate twin analysis, including ACE and ADE-s models
#
# Project: ADHD and hypomania
#
# Data: CATSS-9/12, CATSS-15, CATSS-18
#
# Author: Mark Taylor
#
# Date: 2018-08-17
#-----

setwd('Z:/ADHD_Mania/Data')
list.files()

require(OpenMx)
source('miFunctions.R') # package of functions by Hermine Maes

# import and check data:

data <- read.csv(file='clean data.csv', header=T, sep=',')
names(data); dim(data); is.data.frame(data)

# perform exclusions:

data <- subset(data, bestzyg!=3) # exclude unknown zygosity
data <- subset(data, brain1==0&brain2==0&kromosom1==0&kromosom2==0) # general exclusions
data <- data[!is.na(data$yob), ] # missing covariate information
dim(data) # check remaining numbers

##### ADHD and hypomania #####

### prepare data:

Vars <- c('adhd_p12', 'adhd_p15', 'mania_p15', 'adhd_p18', 'mania_p18') # variables to analyze
nv <- 5 # number of phenotypes
ntv <- nv*2 # total number of variables
selVars <- paste(Vars, c(rep(1, nv), rep(2, nv)), sep='') # variable names

covVars <- 'yob' # covariate (year of birth)

# create subsets of data for each sex/zygosity group for analysis

mzf <- subset(data, bestzyg==1&sex1==2, c(selVars, covVars))
dzf <- subset(data, bestzyg==2&sex1==2, c(selVars, covVars))
mzm <- subset(data, bestzyg==1&sex1==1, c(selVars, covVars))
dzm <- subset(data, bestzyg==2&sex1==1, c(selVars, covVars))
dzos <- subset(data, bestzyg==4, c(selVars, covVars))
### fit a fully saturated model of the observed data

# starting values for the covariances (starting with 1 for variances and 0.5 for covariances):

svCov <- c(1, rep(.5, 9), 1, rep(.5, 8), 1, rep(.5, 7), 1, rep(.5, 6), 1, rep(.5, 5), 1, rep(.5, 4), 1, rep(.5, 3),
        1, rep(.5, 2), 1, .5, 1)

# matrices for means:

meanMZF <- mxMatrix(type='Full', nrow=1, ncol=ntv, free=T, values=.001, labels=labFull('m_mzf', 1, ntv),
          name='mMZF')
meanDZF <- mxMatrix(type='Full', nrow=1, ncol=ntv, free=T, values=.001, labels=labFull('m_dzf', 1, ntv),
          name='mDZF')
```

© 2019 Hosang GM et al. *JAMA Psychiatry.*

```
meanMZM <- mxMatrix(type='Full', nrow=1, ncol=ntv, free=T, values=.001, labels=labFull('m_mzm', 1,
        ntv), name='mMZM')
meanDZM <- mxMatrix(type='Full', nrow=1, ncol=ntv, free=T, values=.001, labels=labFull('m_dzm', 1,
        ntv), name='mDZM')
meanDZOS <- mxMatrix(type='Full', nrow=1, ncol=ntv, free=T, values=.001, labels=labFull('m_dzos', 1, ,
        ntv), name='mDZOS')

# matrices for the effects of the covariates:

beta <- mxMatrix(type='Full', nrow=5, ncol=1, free=T, values=0, label=labFull('beta_yob', 5, 1),
        name='Beta')
cov <- mxMatrix(type='Full', nrow=1, ncol=1, free=F, labels=c('data.yob'), name='Cov')

# calculate means, adjusted for effect of year of birth:

expMeanMZF <- mxAlgebra(mMZF+cbind(t(Beta%*%Cov), t(Beta%*%Cov)), name='ExpMeanMZF')
expMeanDZF <- mxAlgebra(mDZF+cbind(t(Beta%*%Cov), t(Beta%*%Cov)), name='ExpMeanDZF')
expMeanMZM <- mxAlgebra(mMZM+cbind(t(Beta%*%Cov), t(Beta%*%Cov)), name='ExpMeanMZM')
expMeanDZM <- mxAlgebra(mDZM+cbind(t(Beta%*%Cov), t(Beta%*%Cov)), name='ExpMeanDZM')
expMeanDZOS <- mxAlgebra(mDZOS+cbind(t(Beta%*%Cov), t(Beta%*%Cov)), name='ExpMeanDZOS')

# variance/covariance for each sex/zygosity group:

expCovMZF <- mxMatrix(type='Symm', nrow=ntv, ncol=ntv, free=T, values=svCov,
        label=labSymm('mzf_cov', ntv), name='ExpCovMZF')
expCovDZF <- mxMatrix(type='Symm', nrow=ntv, ncol=ntv, free=T, values=svCov,
        label=labSymm('dzf_cov', ntv), name='ExpCovDZF')
expCovMZM <- mxMatrix(type='Symm', nrow=ntv, ncol=ntv, free=T, values=svCov,
        label=labSymm('mzm_cov', ntv),  name='ExpCovMZM')
expCovDZM <- mxMatrix(type='Symm', nrow=ntv, ncol=ntv, free=T, values=svCov,
        label=labSymm('dzm_cov', ntv),  name='ExpCovDZM')
expCovDZOS <- mxMatrix(type='Symm', nrow=ntv, ncol=ntv, free=T, values=svCov,
        label=labSymm('dzos_cov', ntv),  name='ExpCovDZOS')


# observed data for each sex/zygosity group:

dataMZF <- mxData(mzf, type='raw')
dataDZF <- mxData(dzf, type='raw')
dataMZM <- mxData(mzm, type='raw')
dataDZM <- mxData(dzm, type='raw')
dataDZOS <- mxData(dzos, type='raw')

# model objectives:

objMZF <- mxExpectationNormal(covariance='ExpCovMZF', means='ExpMeanMZF', dimnames=selVars)
objDZF <- mxExpectationNormal(covariance='ExpCovDZF', means='ExpMeanDZF', dimnames=selVars)
objMZM <- mxExpectationNormal(covariance='ExpCovMZM', means='ExpMeanMZM',
        dimnames=selVars)
objDZM <- mxExpectationNormal(covariance='ExpCovDZM', means='ExpMeanDZM', dimnames=selVars)
objDZOS <- mxExpectationNormal(covariance='ExpCovDZOS', means='ExpMeanDZOS',
        dimnames=selVars)
funcML <- mxFitFunctionML() # specify maximum likelihood estimation

# data groups:

modelMZF <- mxModel('MZF', meanMZF, beta, cov, expMeanMZF, expCovMZF, dataMZF, objMZF,
        funcML)
modelDZF <- mxModel('DZF', meanDZF, beta, cov, expMeanDZF, expCovDZF, dataDZF, objDZF, funcML)
modelMZM <- mxModel('MZM', meanMZM, beta, cov, expMeanMZM, expCovMZM, dataMZM, objMZM,
        funcML)
modelDZM <- mxModel('DZM', meanDZM, beta, cov, expMeanDZM, expCovDZM, dataDZM, objDZM,
        funcML)
```

© 2019 Hosang GM et al. *JAMA Psychiatry.*

```
modelDZOS <- mxModel('DZOS', meanDZOS, beta, cov, expMeanDZOS, expCovDZOS, dataDZOS,
            objDZOS, funcML)

# combine the groups:

multi <- mxFitFunctionMultigroup(c('MZF', 'DZF', 'MZM', 'DZM', 'DZOS'))

# specify model:

satModel <- mxModel('FullSat', modelMZF, modelDZF, modelMZM, modelDZM, modelDZOS, multi)

# fit the model:

SatModel <- mxTryHard(satModel)
(sumSat <- summary(SatModel)) # print summary of the model



### ACE Cholesky decomposition:

# note: model assumes no qualitative sex limitation, based on univariate model fitting

# starting values for the path coefficients (starting at 1 for variance and .5 for covariance):

svPa <- c(1, .5, .5, .5, .5, 1, .5, .5, .5, 1, .5, .5, 1, .5, 1)

# path coefficients:

pathAf <- mxMatrix(type='Lower', nrow=nv, ncol=nv, free=T, values=svPa, labels=labLower('af', nv),
            name='af')
pathCf <- mxMatrix(type='Lower', nrow=nv, ncol=nv, free=T, values=svPa, labels=labLower('cf', nv),
             name='cf')
pathEf <- mxMatrix(type='Lower', nrow=nv, ncol=nv, free=T, values=svPa, labels=labLower('ef', nv),
            name='ef')

pathAm <- mxMatrix(type='Lower', nrow=nv, ncol=nv, free=T, values=svPa, labels=labLower('am', nv),
            name='am')
pathCm <- mxMatrix(type='Lower', nrow=nv, ncol=nv, free=T, values=svPa, labels=labLower('cm', nv),
            name='cm')
pathEm <- mxMatrix(type='Lower', nrow=nv, ncol=nv, free=T, values=svPa, labels=labLower('em', nv),
            name='em')

# variance components and total variance:

varAf <- mxAlgebra(af%*%t(af), name='Af')
varCf <- mxAlgebra(cf%*%t(cf), name='Cf')
varEf <- mxAlgebra(ef%*%t(ef), name='Ef')

varAm <- mxAlgebra(am%*%t(am), name='Am')
varCm <- mxAlgebra(cm%*%t(cm), name='Cm')
varEm <- mxAlgebra(em%*%t(em), name='Em')

varPf <- mxAlgebra(Af+Cf+Ef, name='Vf')
varPm <- mxAlgebra(Am+Cm+Em, name='Vm')

# calculate squared standardized components (i.e. proportions to report in the paper):

matI <- mxMatrix(type='Iden', nrow=nv, ncol=nv, name='I')
isdf <- mxAlgebra(solve(sqrt(I*Vf)), name='iSDf')
isdm <- mxAlgebra(solve(sqrt(I*Vm)), name='iSDm')

cholAf <- mxAlgebra((iSDf%*%af)^2, name='CholAf')
cholCf <- mxAlgebra((iSDf%*%cf)^2, name='CholCf')
```

© 2019 Hosang GM et al. *JAMA Psychiatry.*

```
cholEf <- mxAlgebra((iSDf%*%ef)^2, name='CholEf')

cholAm <- mxAlgebra((iSDm%*%am)^2, name='CholAm')
cholCm <- mxAlgebra((iSDm%*%cm)^2, name='CholCm')
cholEm <- mxAlgebra((iSDm%*%em)^2, name='CholEm')

# means and covariates:

meanF <- mxMatrix(type='Full', nrow=1, ncol=ntv, free=T, values=.001, labels=labFull('mf', 1, nv), name='Mf')
meanM <- mxMatrix(type='Full', nrow=1, ncol=ntv, free=T, values=.001, labels=labFull('mm', 1, nv),
name='Mm')
meanOS <- mxMatrix(type='Full', nrow=1, ncol=ntv, free=T, values=.001, labels=c(labFull('mf', 1, nv),
                                                         labFull('mm', 1, nv)), name='Mos')

beta <- mxMatrix(type='Full', nrow=5, ncol=1, free=T, values=0, labels=labFull('beta_age', 5, 1),
name='Beta')
cov <- mxMatrix(type='Full', nrow=1, ncol=1, free=F, labels=('data.yob'), name='Cov')

expMeanF <- mxAlgebra(Mf+cbind(t(Beta%*%Cov), t(Beta%*%Cov)), name='ExpMeanF')
expMeanM <- mxAlgebra(Mm+cbind(t(Beta%*%Cov), t(Beta%*%Cov)), name='ExpMeanM')
expMeanOS <- mxAlgebra(Mos+cbind(t(Beta%*%Cov), t(Beta%*%Cov)), name='ExpMeanOS')

# matrix to show variance components as proportions:

rowLabs <- c(rep('VC', nv))
colLabs <- rep(c('A', 'C', 'E', 'A/V', 'C/V', 'E/V'), each=nv)

estVCf <- mxAlgebra(cbind(Af, Cf, Ef, Af/Vf, Cf/Vf, Ef/Vf), name='EstVCf', dimnames=list(rowLabs,
        colLabs))
estVCm <- mxAlgebra(cbind(Am, Cm, Em, Am/Vm, Cm/Vm, Em/Vm), name='EstVCm',
        dimnames=list(rowLabs, colLabs))

# expected variance/covariance:

expCovMZF <- mxAlgebra(rbind(cbind(Vf, Af+Cf),
                                   cbind(Af+Cf, Vf)), name='ExpCovMZF')
expCovDZF <- mxAlgebra(rbind(cbind(Vf, 0.5%x%Af+Cf),
                                   cbind(0.5%x%Af+Cf, Vf)), name='ExpCovDZF')
expCovMZM <- mxAlgebra(rbind(cbind(Vm, Am+Cm),
                                    cbind(Am+Cm, Vm)), name='ExpCovMZM')
expCovDZM <- mxAlgebra(rbind(cbind(Vm, 0.5%x%Am+Cm),
                                    cbind(0.5%x%Am+Cm, Vm)), name='ExpCovDZM')
expCovDZOS <- mxAlgebra(rbind(cbind(Vf, 0.5%x%(af%*%t(am))+cf%*%t(cm)),
                                    cbind(0.5%x%(am%*%t(af))+cm%*%t(cf), Vm)), name='ExpCovDZOS')
 # genetic covariance fixed to 0.5 in OS pairs, assuming no qualitative sex limitation

# observed data:

dataMZF <- mxData(mzf, type='raw')
dataDZF <- mxData(dzf, type='raw')
dataMZM <- mxData(mzm, type='raw')
dataDZM <- mxData(dzm, type='raw')
dataDZOS <- mxData(dzos, type='raw')

# objectives:

objMZF <- mxExpectationNormal(covariance='ExpCovMZF', means='ExpMeanF', dimnames=selVars)
objDZF <- mxExpectationNormal(covariance='ExpCovDZF', means='ExpMeanF', dimnames=selVars)
objMZM <- mxExpectationNormal(covariance='ExpCovMZM', means='ExpMeanM', dimnames=selVars)
objDZM <- mxExpectationNormal(covariance='ExpCovDZM', means='ExpMeanM', dimnames=selVars)
objDZOS <- mxExpectationNormal(covariance='ExpCovDZOS', means='ExpMeanOS', dimnames=selVars)
funcML <- mxFitFunctionML()
```

© 2019 Hosang GM et al. *JAMA Psychiatry.*

data groups:

```
covVars <- list(beta, cov) # covariates
parsF <- list(pathAf, pathCf, pathEf, varAf, varCf, varEf, varPf, matI, isdf, cholAf, cholCf, cholEf, meanF,
        expMeanF)
parsM <- list(pathAm, pathCm, pathEm, varAm, varCm, varEm, varPm, matI, isdm, cholAm, cholCm,
        cholEm, meanM, expMeanM)

modelMZF <- mxModel('MZF', covVars, parsF, estVCf, expCovMZF, dataMZF, objMZF, funcML)
modelDZF <- mxModel('DZF', covVars, parsF, estVCf, expCovDZF, dataDZF, objDZF, funcML)
modelMZM <- mxModel('MZM', covVars, parsM, estVCm, expCovMZM, dataMZM, objMZM, funcML)
modelDZM <- mxModel('DZM', covVars, parsM, estVCm, expCovDZM, dataDZM, objDZM, funcML)
modelDZOS <- mxModel('DZOS', covVars, parsM, parsF, meanOS, expMeanOS, estVCm, estVCf,
        expCovDZOS, dataDZOS, objDZOS, funcML)

# combine groups:

multi <- mxFitFunctionMultigroup(c('MZF', 'DZF', 'MZM', 'DZM', 'DZOS'))

# confidence intervals:

ci <- mxCI(c('MZF.EstVCf', 'MZM.EstVCm', 'MZF.CholAf', 'MZF.CholCf', 'MZF.CholEf', 'MZM.CholAm',
        'MZM.CholCm', 'MZM.CholEm'))

# build the model:

modelACE <- mxModel('ACE', modelMZF, modelDZF, modelMZM, modelDZM, modelDZOS, multi, ci)

# fit the model:

ModelACE <- mxTryHard(modelACE, intervals=F)
sumACE <-summary(ModelACE); sumACE



### ADE-s model:

# logicals for the sibling interaction paths (to ‘zoom in’ on main parameters of interest)

sibLog <- c(rep(F, 5), T, rep(F, 10), T, rep(F, 21), T, rep(F, 11), T, rep(F, 10), T, rep(F, 21), T, rep(F, 16))

# path coefficients:

pathAf <- mxMatrix(type='Lower', nrow=nv, ncol=nv, free=T, values=svPa, labels=labLower('af', nv),
        name='af')
pathDf <- mxMatrix(type='Lower', nrow=nv, ncol=nv, free=T, values=svPa, labels=labLower('df', nv),
        name='df')
pathEf <- mxMatrix(type='Lower', nrow=nv, ncol=nv, free=T, values=svPa, labels=labLower('ef', nv),
        name='ef')

pathAm <- mxMatrix(type='Lower', nrow=nv, ncol=nv, free=T, values=svPa, labels=labLower('am', nv),
        name='am')
pathDm <- mxMatrix(type='Lower', nrow=nv, ncol=nv, free=T, values=svPa, labels=labLower('dm', nv),
        name='dm')
pathEm <- mxMatrix(type='Lower', nrow=nv, ncol=nv, free=T, values=svPa, labels=labLower('em', nv),
        name='em')

# variance components and total variance:

varAf <- mxAlgebra(af%*%t(af), name='Af')
varDf <- mxAlgebra(df%*%t(df), name='Df')
varEf <- mxAlgebra(ef%*%t(ef), name='Ef')
```

© 2019 Hosang GM et al. *JAMA Psychiatry.*

```
varAm <- mxAlgebra(am%*%t(am), name='Am')
varDm <- mxAlgebra(dm%*%t(dm), name='Dm')
varEm <- mxAlgebra(em%*%t(em), name='Em')

varPf <- mxAlgebra(Af+Df+Ef, name='Vf')
varPm <- mxAlgebra(Am+Dm+Em, name='Vm')

# calculate squared standardized pathways (i.e. proportion of each trait due to each component)

matI <- mxMatrix(type='Iden', nrow=nv, ncol=nv, name='I')
isdf <- mxAlgebra(solve(sqrt(I*Vf)), name='iSDf')
isdm <- mxAlgebra(solve(sqrt(I*Vm)), name='iSDm')

cholAf <- mxAlgebra((iSDf%*%af)^2, name='CholAf')
cholDf <- mxAlgebra((iSDf%*%df)^2, name='CholDf')
cholEf <- mxAlgebra((iSDf%*%ef)^2, name='CholEf')

cholAm <- mxAlgebra((iSDm%*%am)^2, name='CholAm')
cholDm <- mxAlgebra((iSDm%*%dm)^2, name='CholDm')
cholEm <- mxAlgebra((iSDm%*%em)^2, name='CholEm')

# sibling interaction pathways:

matI2 <- mxMatrix(type='Iden', nrow=ntv, ncol=ntv, name='I2')

sibF <- mxMatrix(type='Symm', nrow=ntv, ncol=ntv, free=sibLog, values=0, labels=labSymm('bf', ntv),
        lbound=-.9999,  ubound=.9999, name='bf')
sibM <- mxMatrix(type='Symm', nrow=ntv, ncol=ntv, free=sibLog, values=0, labels=labSymm('bm', ntv),
        lbound=-.9999, ubound=.9999, name='bm')
sibOS <- mxMatrix(type='Symm', nrow=ntv, ncol=ntv, free=sibLog, values=0, labels=labSymm('bos', ntv),
        lbound=-.9999, ubound=.9999, name='bos')

# means and covariates:

meanF <- mxMatrix(type='Full', nrow=1, ncol=ntv, free=T, values=.001, labels=labFull('mf', 1, nv),
        name='Mf')
meanM <- mxMatrix(type='Full', nrow=1, ncol=ntv, free=T, values=.001, labels=labFull('mm', 1, nv),
        name='Mm')
meanOS <- mxMatrix(type='Full', nrow=1, ncol=ntv, free=T, values=.001, labels=c(labFull('mf', 1, nv),
                    labFull('mm', 1, nv)), name='Mos')

beta <- mxMatrix(type='Full', nrow=5, ncol=1, free=T, values=0, labels=labFull('beta_age', 5, 1),
        name='Beta')
cov <- mxMatrix(type='Full', nrow=1, ncol=1, free=F, labels=('data.yob'), name='Cov')

expMeanF <- mxAlgebra(Mf+cbind(t(Beta%*%Cov), t(Beta%*%Cov)), name='ExpMeanF')
expMeanM <- mxAlgebra(Mm+cbind(t(Beta%*%Cov), t(Beta%*%Cov)), name='ExpMeanM')
expMeanOS <- mxAlgebra(Mos+cbind(t(Beta%*%Cov), t(Beta%*%Cov)), name='ExpMeanOS')

# matrix of variance components:

rowLabs <- c(rep('VC', nv))
colLabs <- rep(c('A', 'D', 'E', 'A/V', 'D/V', 'E/V'), each=nv)

estVCf <- mxAlgebra(cbind(Af, Df, Ef, Af/Vf, Df/Vf, Ef/Vf), name='EstVCf', dimnames=list(rowLabs, colLabs))
estVCm <- mxAlgebra(cbind(Am, Dm, Em, Am/Vm, Dm/Vm, Em/Vm), name='EstVCm',
dimnames=list(rowLabs, colLabs))

# expected variance/covariance (again assuming no qualitative sex limitation):

expCovMZF <- mxAlgebra(solve(I2-bf)%&%(rbind(cbind(Vf, Af+Df),
                                                  cbind(Af+Df, Vf))), name='ExpCovMZF')
expCovDZF <- mxAlgebra(solve(I2-bf)%&%(rbind(cbind(Vf, 0.5%x%Af+0.25%x%Df),
```

© 2019 Hosang GM et al. *JAMA Psychiatry.*

```
                                        cbind(0.5%x%Af+0.25%x%Df, Vf))),
name='ExpCovDZF')
expCovMZM <- mxAlgebra(solve(I2-bm)%&%(rbind(cbind(Vm, Am+Dm),
                                          cbind(Am+Dm, Vm))), name='ExpCovMZM')

expCovDZM <- mxAlgebra(solve(I2-bm)%&%(rbind(cbind(Vm, 0.5%x%Am+0.25%x%Dm),
                                     cbind(0.5%x%Am+0.25%x%Dm, Vm))),
name='ExpCovDZM')
expCovDZOS <- mxAlgebra(solve(I2-bos)%&%(rbind(cbind(Vf,
                                          0.5%x%(af%*%t(am))+0.25%x%(df%*%t(dm))),
                    cbind(0.5%x%(am%*%t(af))+0.25%x%(dm%*%t(df)), Vm))),
name='ExpCovDZOS')

# observed data:

dataMZF <- mxData(mzf, type='raw')
dataDZF <- mxData(dzf, type='raw')
dataMZM <- mxData(mzm, type='raw')
dataDZM <- mxData(dzm, type='raw')
dataDZOS <- mxData(dzos, type='raw')

# objectives:

objMZF <- mxExpectationNormal(covariance='ExpCovMZF', means='ExpMeanF', dimnames=selVars)
objDZF <- mxExpectationNormal(covariance='ExpCovDZF', means='ExpMeanF', dimnames=selVars)
objMZM <- mxExpectationNormal(covariance='ExpCovMZM', means='ExpMeanM', dimnames=selVars)
objDZM <- mxExpectationNormal(covariance='ExpCovDZM', means='ExpMeanM', dimnames=selVars)
objDZOS <- mxExpectationNormal(covariance='ExpCovDZOS', means='ExpMeanOS', dimnames=selVars)
funcML <- mxFitFunctionML()

# data groups:

covVars <- list(beta, cov)
parsF <- list(pathAf, pathDf, pathEf, varAf, varDf, varEf, varPf, matI, isdf, cholAf, cholDf, cholEf, matI2,
      sibF, meanF, expMeanF)
parsM <- list(pathAm, pathDm, pathEm, varAm, varDm, varEm, varPm, matI, isdm, cholAm, cholDm,
cholEm, matI2, sibM,
       meanM, expMeanM)

modelMZF <- mxModel('MZF', covVars, parsF, estVCf, expCovMZF, dataMZF, objMZF, funcML)
modelDZF <- mxModel('DZF', covVars, parsF, estVCf, expCovDZF, dataDZF, objDZF, funcML)
modelMZM <- mxModel('MZM', covVars, parsM, estVCm, expCovMZM, dataMZM, objMZM, funcML)
modelDZM <- mxModel('DZM', covVars, parsM, estVCm, expCovDZM, dataDZM, objDZM, funcML)
modelDZOS <- mxModel('DZOS', covVars, parsF, parsM, meanOS, expMeanOS, estVCf, estVCm,
      expCovDZOS, dataDZOS, objDZOS, sibOS, funcML)

# combine the groups:

multi <- mxFitFunctionMultigroup(c('MZF', 'DZF', 'MZM', 'DZM', 'DZOS'))

# confidence intervals:

ci <- mxCI(c('MZF.EstVCf', 'MZM.EstVCm', 'MZF.bf', 'MZM.bm', 'DZOS.bos', 'MZF.CholAf', 'MZF.CholDf',
      'MZF.CholEf', 'MZM.CholAm', 'MZM.CholDm', 'MZM.CholEm'))

# build the model:

modelADEs <- mxModel('ADEs', modelMZF, modelDZF, modelMZM, modelDZM, modelDZOS, multi, ci)

# fit the model:

ModelADEs <- mxTryHard(modelADEs, intervals=F)
sumADEs <- summary(ModelADEs); sumADEs
```

© 2019 Hosang GM et al. *JAMA Psychiatry.*

```
### joint ACE/ADE model

## Note on this model: since hypomania at age 15 shows some C, but no other measure does, this model ##
estimates C for that phenotype, but D/s for everything else. As a consequence, C and D do not
## influence the covariance between phenotypes, only variance in each phenotype.

# logicals and starting values for b, C and D path coefficients:

bPathLog <- c(rep(F, 5), T, rep(F, 10), T, rep(F, 10), T, rep(F, 22), T, rep(F, 10), T, rep(F, 10), T, rep(F, 27))
cPathLog <- c(rep(F, 9), T, rep(F, 4), T)
dPathLog <- c(T, rep(F, 4), T, rep(F, 6), T, rep(F, 2))

svC <- c(rep(0, 9), 1, rep(0, 4), 1)
svD <- c(1, rep(0, 4), 1, rep(0, 6), 1, rep(0, 2))

# path coefficients (C fixed to zero for ADHD, D fixed to zero for hypomania):

pathAf <- mxMatrix(type='Lower', nrow=nv, ncol=nv, free=T, values=svPa, labels=labLower('af', nv),
        name='af')
pathDf <- mxMatrix(type='Lower', nrow=nv, ncol=nv, free=dPathLog, values=svD, labels=labLower('df',
        nv), name='df')
pathCf <- mxMatrix(type='Lower', nrow=nv, ncol=nv, free=cPathLog, values=svC, labels=labLower('cf',
        nv), name='cf')
pathEf <- mxMatrix(type='Lower', nrow=nv, ncol=nv, free=T, values=svPa, labels=labLower('ef', nv),
        name='ef')

pathAm <- mxMatrix(type='Lower', nrow=nv, ncol=nv, free=T, values=svPa, labels=labLower('am', nv),
        name='am')
pathDm <- mxMatrix(type='Lower', nrow=nv, ncol=nv, free=dPathLog, values=svD, labels=labLower('dm',
        nv), name='dm')
pathCm <- mxMatrix(type='Lower', nrow=nv, ncol=nv, free=cPathLog, values=svC, labels=labLower('cm',
        nv), name='cm')
pathEm <- mxMatrix(type='Lower', nrow=nv, ncol=nv, free=T, values=svPa, labels=labLower('em', nv),
        name='em')

# variance components and total variance:

varAf <- mxAlgebra(af%*%t(af), name='Af')
varDf <- mxAlgebra(df%*%t(df), name='Df')
varCf <- mxAlgebra(cf%*%t(cf), name='Cf')
varEf <- mxAlgebra(ef%*%t(ef), name='Ef')

varAm <- mxAlgebra(am%*%t(am), name='Am')
varDm <- mxAlgebra(dm%*%t(dm), name='Dm')
varCm <- mxAlgebra(cm%*%t(cm), name='Cm')
varEm <- mxAlgebra(em%*%t(em), name='Em')

varPf <- mxAlgebra(Af+Df+Cf+Ef, name='Vf')
varPm <- mxAlgebra(Am+Dm+Cm+Em, name='Vm')

# calculate standardized, squared pathways

matI <- mxMatrix(type='Iden', nrow=nv, ncol=nv, name='I')
isdf <- mxAlgebra(solve(sqrt(I*Vf)), name='iSDf')
isdm <- mxAlgebra(solve(sqrt(I*Vm)), name='iSDm')

cholAf <- mxAlgebra((iSDf%*%af)^2, name='CholAf')
cholDf <- mxAlgebra((iSDf%*%df)^2, name='CholDf')
cholCf <- mxAlgebra((iSDf%*%cf)^2, name='CholCf')
cholEf <- mxAlgebra((iSDf%*%ef)^2, name='CholEf')

cholAm <- mxAlgebra((iSDm%*%am)^2, name='CholAm')
```

© 2019 Hosang GM et al. *JAMA Psychiatry.*

```
cholDm <- mxAlgebra((iSDm%*%dm)^2, name='CholDm')
cholCm <- mxAlgebra((iSDm%*%cm)^2, name='CholCm')
cholEm <- mxAlgebra((iSDm%*%em)^2, name='CholEm')

# sibling interaction paths:

matI2 <- mxMatrix(type='Iden', nrow=ntv, ncol=ntv, name='I2')

pathBf <- mxMatrix(type='Symm', nrow=ntv, ncol=ntv, free=sibLog, values=0, label=labSymm('bf', ntv),
        lbound=-.9999, ubound=.9999, name='bf')
pathBm <- mxMatrix(type='Symm', nrow=ntv, ncol=ntv, free=sibLog, values=0, label=labSymm('bm', ntv),
        lbound=-.9999, ubound=.9999, name='bm')
pathBos <- mxMatrix(type='Symm', nrow=ntv, ncol=ntv, free=sibLog, values=0, label=labSymm('bos',
        ntv), lbound=-.9999, ubound=.9999, name='bos')

# means and covariates:

meanF <- mxMatrix(type='Full', nrow=1, ncol=ntv, free=T, values=.001, labels=labFull('mf', 1, nv),
        name='Mf')
meanM <- mxMatrix(type='Full', nrow=1, ncol=ntv, free=T, values=.001, labels=labFull('mm', 1, nv),
        name='Mm')
meanOS <- mxMatrix(type='Full', nrow=1, ncol=ntv, free=T, values=.001, labels=c(labFull('mf', 1, nv),
          labFull('mm', 1, nv)), name='Mos')

beta <- mxMatrix(type='Full', nrow=5, ncol=1, free=T, values=0, labels=labFull('beta_age', 5, 1),
name='Beta')
cov <- mxMatrix(type='Full', nrow=1, ncol=1, free=F, labels=('data.yob'), name='Cov')

expMeanF <- mxAlgebra(Mf+cbind(t(Beta%*%Cov), t(Beta%*%Cov)), name='ExpMeanF')
expMeanM <- mxAlgebra(Mm+cbind(t(Beta%*%Cov), t(Beta%*%Cov)), name='ExpMeanM')
expMeanOS <- mxAlgebra(Mos+cbind(t(Beta%*%Cov), t(Beta%*%Cov)), name='ExpMeanOS')

# matrix of variance components:

rowVC <- c(rep('VC', nv))
colVC <- rep(c('A', 'D', 'C', 'E', 'A/V', 'D/V', 'C/V', 'E/V'), each=nv)

estVCf <- mxAlgebra(cbind(Af, Df, Cf, Ef, Af/Vf, Df/Vf, Cf/Vf, Ef/Vf), name='EstVCf', dimnames=list(rowVC,
colVC))
estVCm <- mxAlgebra(cbind(Am, Dm, Cm, Em, Am/Vm, Dm/Vm, Cm/Vm, Em/Vm), name='EstVCm',
dimnames=list(rowVC, colVC))

# expected variance/covariance:

expCovMZF <- mxAlgebra(solve(I2-bf)%&%(rbind(cbind(Vf, Af+Df+Cf),
                              cbind(Af+Df+Cf, Vf))), name='ExpCovMZF')
expCovDZF <- mxAlgebra(solve(I2-bf)%&%(rbind(cbind(Vf, 0.5%x%Af+0.25%x%Df+Cf),
                              cbind(0.5%x%Af+0.25%x%Df+Cf, Vf))), name='ExpCovDZF')
expCovMZM <- mxAlgebra(solve(I2-bm)%&%(rbind(cbind(Vm, Am+Dm+Cm),
                              cbind(Am+Dm+Cm, Vm))), name='ExpCovMZM')
expCovDZM <- mxAlgebra(solve(I2-bm)%&%(rbind(cbind(Vm,
                      0.5%x%Am+0.25%x%Dm+Cm),
                              cbind(0.5%x%Am+0.25%x%Dm+Cm, Vm))), name='ExpCovDZM')
expCovDZOS <- mxAlgebra(solve(I2-bos)%&%(rbind(cbind(Vf,
                                0.5%x%(af%*%t(am))+0.25%x%(df%*%t(dm))+cf%*%t(cm)),
                               cbind(0.5%x%(am%*%t(af))+0.25%x%(dm%*%t(df))+cm%*%t(cf), Vm))),
              name='ExpCovDZOS')

# data:

dataMZF <- mxData(mzf, type='raw')
dataDZF <- mxData(dzf, type='raw')
dataMZM <- mxData(mzm, type='raw')
```

© 2019 Hosang GM et al. *JAMA Psychiatry.*

```
dataDZM <- mxData(dzm, type='raw')
dataDZOS <- mxData(dzos, type='raw')

# objectives:

objMZF <- mxExpectationNormal(covariance='ExpCovMZF', means='ExpMeanF', dimnames=selVars)
objDZF <- mxExpectationNormal(covariance='ExpCovDZF', means='ExpMeanF', dimnames=selVars)
objMZM <- mxExpectationNormal(covariance='ExpCovMZM', means='ExpMeanM', dimnames=selVars)
objDZM <- mxExpectationNormal(covariance='ExpCovDZM', means='ExpMeanM', dimnames=selVars)
objDZOS <- mxExpectationNormal(covariance='ExpCovDZOS', means='ExpMeanOS', dimnames=selVars)
funcML <- mxFitFunctionML()

# data groups:

covVars <- list(beta, cov)
parsF <- list(pathAf, pathDf, pathCf, pathEf, varAf, varDf, varCf, varEf, varPf, matI, isdf, cholAf, cholDf,
        cholCf, cholEf, matI2, pathBf, meanF, expMeanF)
parsM <- list(pathAm, pathDm, pathCm, pathEm, varAm, varDm, varCm, varEm, varPm, matI, isdm,
        cholAm, cholDm, cholCm, cholEm, matI2, pathBm, meanM, expMeanM)

modelMZF <- mxModel('MZF', covVars, parsF, estVCf, expCovMZF, dataMZF, objMZF, funcML)
modelDZF <- mxModel('DZF', covVars, parsF, estVCf, expCovDZF, dataDZF, objDZF, funcML)
modelMZM <- mxModel('MZM', covVars, parsM, estVCm, expCovMZM, dataMZM, objMZM, funcML)
modelDZM <- mxModel('DZM', covVars, parsM, estVCm, expCovDZM, dataDZM, objDZM, funcML)
modelDZOS <- mxModel('DZOS', covVars, parsF, parsM, meanOS, expMeanOS, estVCf, estVCm, matI2,
                        pathBos, expCovDZOS, dataDZOS, objDZOS, funcML)

# combine groups:

multi <- mxFitFunctionMultigroup(c('MZF', 'DZF', 'MZM', 'DZM', 'DZOS'))

# confidence intervals:

ci <- mxCI(c('MZF.EstVCf', 'MZF.CholAf', 'MZF.CholDf', 'MZF.CholCf', 'MZF.CholEf', 'MZF.bf',
             'MZM.EstVCm',  'MZM.CholAm', 'MZM.CholDm', 'MZM.CholCm', 'MZM.CholEm',
             'MZM.bm', 'DZOS.bos'))

# combine everything:

modelADCE <- mxModel('ADCE', modelMZF, modelDZF, modelMZM, modelDZM, modelDZOS, multi, ci)

# fit the model:

ModelADCE <- mxTryHard(modelADCE, intervals=F)
sumADCE <- summary(ModelADCE); sumADCE

# check fit statistics:

(Fit <- mxCompare(SatModel, c(ModelACE, ModelADEs, ModelADCE)))



### drop sex differences from the ADCE model:

modelADCEHom <- mxModel(ModelADCE, name='ADCEHom')
modelADCEHom <- omxSetParameters(modelADCEHom, labels=c(labLower('af', nv), labLower('am', nv)),
        free=T,  values=svPa, newlabels=labLower('a', nv))
modelADCEHom <- omxSetParameters(modelADCEHom, labels=c(labLower('df', nv), labLower('dm', nv)),
                free=dPathLog, values=svD, newlabels=labLower('d', nv))
modelADCEHom <- omxSetParameters(modelADCEHom, labels=c(labLower('cf', nv), labLower('cm', nv)),
                free=cPathLog, values=svC, newlabels=labLower('c', nv))
modelADCEHom <- omxSetParameters(modelADCEHom, labels=c(labLower('ef', nv), labLower('em', nv)),
        free=T, values=svPa, newlabels=labLower('e', nv))
```

© 2019 Hosang GM et al. *JAMA Psychiatry.*

```
modelADCEHom <- omxSetParameters(modelADCEHom, labels=c('bf_6_1', 'bf_7_2', 'bf_9_4', 'bm_6_1',
        'bm_7_2', 'bm_9_4'), free=T, values=0, lbound=-.9999, ubound=.9999,
        newlabels=c('b_6_1', 'b_7_2', 'b_9_4'))
ModelADCEHom <- mxTryHard(modelADCEHom, intervals=F)
(sumHom <- summary(ModelADCEHom))
homFit <- mxCompare(ModelADCE, ModelADCEHom)

### drop D:

modelACEs <- mxModel(ModelADCE, name='ACEs')
modelACEs <- omxSetParameters(modelACEs, labels=c(labLower('df', nv), labLower('dm', nv)), free=F,
        values=0)
ModelACEs <- mxTryHard(modelACEs, intervals=T)
(sumACEs <- summary(ModelACEs))
mxCompare(ModelADCE, ModelACEs)

### drop C:

modelADEs <- mxModel(ModelADCE, name='ADEs')
modelADEs <- omxSetParameters(modelADEs, labels=c(labLower('cf', nv), labLower('cm', nv)), free=F,
        values=0)
ModelADEs <- mxTryHard(modelADEs, intervals=F)
(sumADEs <- summary(ModelADEs))
mxCompare(ModelADCE, ModelADEs)

### AE-s model:

modelAEs <- mxModel(ModelADEs, name='AEs')
modelAEs <- omxSetParameters(modelAEs, labels=c(labLower('df', nv), labLower('dm', nv)), free=F,
        values=0)
ModelAEs <- mxTryHard(modelAEs, intervals=F)
(sumAEs <- summary(ModelAEs))
mxCompare(ModelADCE, ModelAEs)


### AE model:

modelAE <- mxModel(ModelAEs, name='AE')
modelAE <- omxSetParameters(modelAE, labels=c(labSymm('bf', ntv), labSymm('bm', ntv),
        labSymm('bos', ntv)), free=F, values=0)
ModelAE <- mxTryHard(modelAE, intervals=F)
(sumAE <- summary(ModelAE))
mxCompare(ModelADCE, ModelAE)

### E model:

modelE <- mxModel(ModelAE, name='E')
modelE <- omxSetParameters(modelE, labels=c(labLower('af', nv), labLower('am', nv)), free=F, values=0)
ModelE <- mxTryHard(modelE, intervals=F)
(sumE <- summary(ModelE))
```

© 2019 Hosang GM et al. *JAMA Psychiatry.*
